# Natural Polymer-Based Hemostatic Hydrogels with Advanced Material and Structural Designs for Functional Applications

**DOI:** 10.3390/pharmaceutics18070820

**Published:** 2026-07-02

**Authors:** Lixin A, Zhaoming Guo, Chen Zhao, Guangyao Li, Xinwen Xu, Yongai Yu, Peng Qu, Qiang Liu

**Affiliations:** 1 Central Hospital of Dalian University of Technology, Dalian 116089, China; 2 Faculty of Medicine, Dalian University of Technology, Dalian 116024, China; 3 School of Chemical Engineering, Marine and Life Sciences, Dalian University of Technology, Panjin 124221, China

**Keywords:** natural polymers, hemostatic hydrogels, material design, structural construction, functionalities

## Abstract

Uncontrolled hemorrhage remains a major challenge in trauma care and surgical interventions, where rapid hemostasis and wound sealing are essential for improving patient survival. Natural polymer-based hydrogels have emerged as promising hemostatic materials owing to their excellent biocompatibility, biodegradability, and biomimetic properties. However, their clinical translation remains limited by insufficient mechanical robustness, wet adhesion, and functional responsiveness. To address these challenges, considerable progress has been achieved through rational material design and structural engineering strategies. Representative natural polymers, particularly polysaccharides and proteins, exhibit distinct physicochemical and biological characteristics that determine their hemostatic mechanisms and design strategies. Based on these material platforms, molecular modification strategies, including charge regulation, hydrophobic modification, and bioactive functionalization, have been widely employed to modulate interfacial interactions, platelet adhesion, coagulation activation, and tissue adhesion. In parallel, advanced structural architectures, such as porous, particulate, fibrous, multicrosslinked/multinetwork, and nanocomposite systems, have significantly enhanced fluid absorption, mechanical resilience, stress dissipation, and hemorrhage sealing efficiency. Beyond conventional hemostasis, increasing efforts have focused on integrating multifunctional properties, including antibacterial activity, inflammatory regulation, oxidative stress modulation, tissue regeneration, dynamic monitoring, and stimuli-responsive behaviors. This review systematically summarizes recent advances in natural polymer-based hemostatic hydrogels from the perspectives of advanced material modification strategies, structural engineering approaches, and functional integration, with particular emphasis on the relationships among material characteristics, interfacial behavior, structural organization, and hemostatic performance. Finally, current challenges and future perspectives for clinical translation are discussed, aiming to provide valuable insights for the rational design and clinical implementation of next-generation hemostatic biomaterials.

## 1. Introduction

Uncontrolled acute bleeding caused by daily accidents, severe traffic trauma, or complex surgical procedures remains one of the leading causes of trauma-related mortality worldwide [1]. Massive blood loss following vascular rupture rapidly disrupts hemodynamic balance and physiological homeostasis. Failure to achieve effective hemostasis leads to insufficient tissue perfusion and persistent hypoxia, forcing cellular metabolism to shift from aerobic to anaerobic pathways. This metabolic transition results in lactic acid accumulation and subsequently induces systemic hypothermia, metabolic acidosis, and coagulopathy, which mutually aggravate each other and ultimately form the highly lethal triad of trauma [2]. Clinical studies indicate that approximately 40% of patients with severe trauma die within 24 h of injury due to hemorrhagic shock or secondary coagulopathy [3], highlighting the critical importance of rapid hemostasis in prehospital emergency care and surgical interventions. Despite substantial advances in hemostatic management, current hemostatic strategies remain inadequate for complex clinical bleeding scenarios. Conventional approaches, such as suturing, tourniquet application, and gauze compression, primarily rely on physical sealing or external compression and are often ineffective for irregular deep wounds or non-compressible hemorrhage in abdominal and pelvic regions [4]. Although commercially available hemostatic materials have improved hemorrhage control to some extent, many still suffer from limited biocompatibility, poor degradation behavior, and unstable interfacial performance under wet and dynamic conditions. For instance, some inorganic hemostatic agents may generate excessive heat during fluid absorption and pose risks of tissue damage or distal embolization [5], whereas synthetic tissue adhesives often exhibit insufficient biodegradability and compromised adhesion in hydrated biointerfaces [6]. Collectively, these limitations substantially restrict the reliable performance of existing hemostatic materials in complex physiological environments. Therefore, the development of advanced hemostatic systems capable of rapid response and stable functionality under wet, dynamic, and high-pressure conditions has become a major focus in biomaterials research.

Among emerging biomaterials, natural polymer-based hydrogels have attracted increasing attention for hemostatic and wound repair applications because of their excellent biocompatibility, biodegradability, low immunogenicity, and structural similarity to the extracellular matrix (ECM) [7]. Representative materials include polysaccharides such as chitosan, cellulose, alginate, hyaluronic acid, and starch, as well as protein-based materials including collagen, gelatin, silk fibroin, fibronectin, and fibrin. These natural polymers possess abundant functional groups, including amino, hydroxyl, and carboxyl moieties, which provide versatile platforms for chemical modification, intermolecular interaction, and biofunctional integration. In addition, their intrinsic biological activities can facilitate cell adhesion, coagulation regulation, inflammatory modulation, and tissue regeneration [8,9]. Nevertheless, despite these advantages, conventional natural polymer-based hydrogels still exhibit limited mechanical robustness, insufficient wet tissue adhesion, rapid degradation, and restricted functionality under complex physiological conditions, thereby hindering their broader clinical translation. To overcome these limitations, substantial efforts have been devoted to the advanced design of natural polymer-based hemostatic hydrogels through material modification and structural engineering strategies [10]. At the material level, increasing attention has been directed toward molecular modification approaches, including charge regulation, hydrophobic modification, dynamic bonding, and bioactive functionalization, to improve blood-material interactions, platelet adhesion, coagulation activation, and tissue adhesion performance. From the structural perspective, diverse engineering approaches, such as porous architectures, particle-based systems, fibrous networks, multicrosslinked/multinetwork systems, and nanocomposite structures, have been developed to regulate fluid transport behavior, stress dissipation pathways, mechanical stability, and rapid wound sealing capability under hemorrhagic conditions. Beyond conventional hemostatic functions, recent advances have further expanded hydrogel systems toward broader functional applications, including coagulation regulation, antimicrobial therapy, anti-inflammatory, tissue regenerative, dynamic monitoring and stimuli-responsive capabilities, thereby enabling more comprehensive regulation of the wound microenvironment and tissue repair processes. Although considerable progress has been achieved, several critical challenges remain unresolved, including raw material variability, biosafety concerns, systemic procoagulant risks, insufficient large-animal validation, difficulties in scalable manufacturing, and the complexity of clinical regulatory approval. Moreover, increasing functional integration frequently introduces additional structural complexity, which may compromise translational feasibility and manufacturing reproducibility. Therefore, achieving a rational balance among material design, structural performance, functional bioactivity, and clinical practicality remains a major objective in the development of next-generation hemostatic hydrogels.

**Figure 1:**
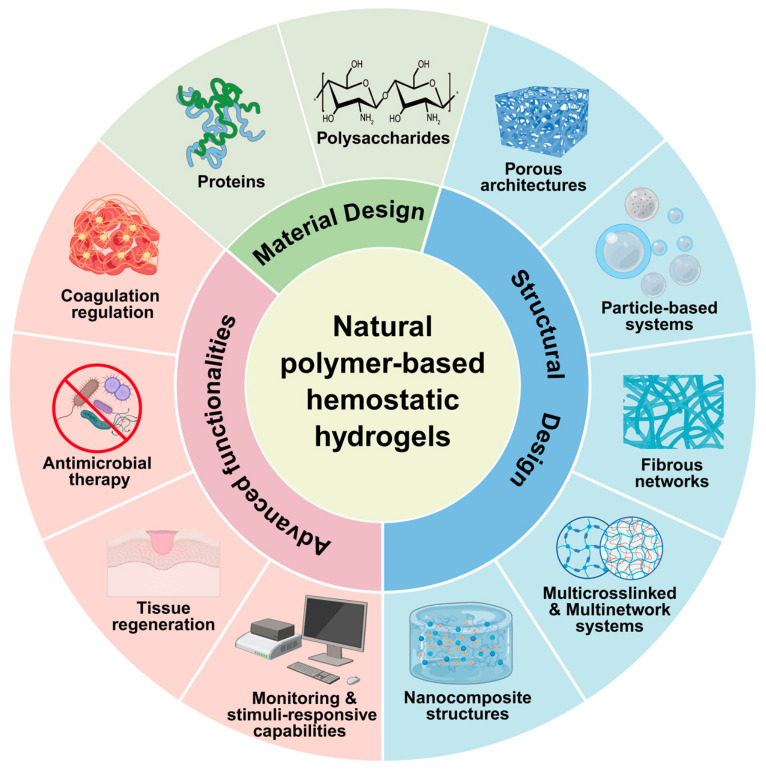
The material design, structural design and advanced functionalities of natural polymer-based hemostatic hydrogels. Asterisks (*) denote the continuation of the polymer backbone.

In this review, recent advances in natural polymer-based hemostatic hydrogels published primarily during the past seven years (2020–2026) are systematically summarized from the perspectives of advanced material molecular engineering, structural design, and functional integration, as illustrated in Figure 1. Representative earlier studies that established key design principles are also discussed where appropriate. Particular emphasis is placed on the relationships among molecular interactions, structural organization, interfacial behavior, and macroscopic hemostatic performance. The advantages and limitations of representative natural polymers and structural architectures are critically discussed, together with emerging functional applications involving coagulation regulation, antibacterial activity, inflammatory regulation, oxidative stress modulation, tissue regeneration, dynamic monitoring and stimuli-responsive behaviors. Finally, current challenges in biosafety, degradation regulation, manufacturing consistency, and clinical translation are discussed. This review aims to provide a framework for the rational design of next-generation natural hemostatic hydrogels with improved performance and translational potential.

## 2. Basic Principles of Hemostasis

### 2.1. Physiological Hemostasis

**Figure 2:**
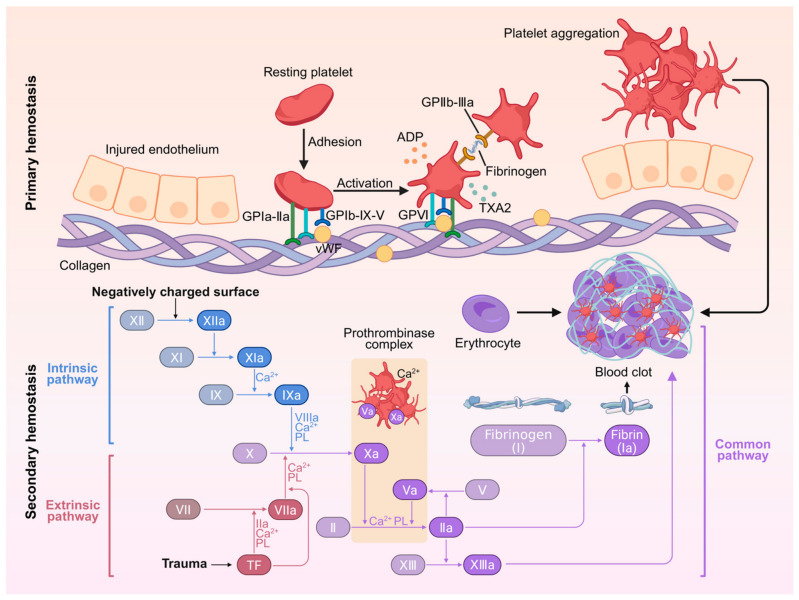
Schematic illustration of the physiologic hemostasis process including primary hemostasis (von Willebrand factor (vWF) and collagen mediated platelet adhesion, activation, and GPIIb-IIIa dependent platelet aggregation into a platelet plug) and secondary hemostasis (the coagulation cascade triggered by tissue factor (TF), assembly of the prothrombinase complex, thrombin (FIIa) generation, fibrin formation, and crosslinking by FXIIIa to form a stable blood clot).

The design of hemostatic materials is fundamentally based on the simulation and regulation of the physiological hemostasis process. Therefore, a comprehensive understanding of the mechanisms underlying blood coagulation under physiological conditions is a critical prerequisite for the rational design and optimization of hemostatic materials. Physiological hemostasis is a highly coordinated protective process by which the body responds to vascular injury and prevents excessive blood loss, primarily through the synergistic actions of the vascular wall, platelets, and the coagulation system. Although physiological hemostasis is conventionally divided into primary hemostasis and secondary hemostasis (Figure 2), these two processes overlap temporally and spatially and function cooperatively throughout coagulation. Upon vascular endothelial injury and exposure of the subendothelial matrix, the hemostatic response is rapidly initiated. Neurogenic reflexes and local humoral factors induce transient vasoconstriction at the injury site, thereby reducing local blood flow and creating favorable conditions for subsequent hemostatic reactions. Simultaneously, circulating platelets are rapidly recruited to the injured region and adhere to exposed collagen through glycoprotein (GP) VI and GP Ia/IIa receptors on the platelet surface. Under high-shear blood flow conditions, the GP Ib-IX-V complex further interacts with von Willebrand factor (vWF), thereby ensuring stable platelet adhesion. Following adhesion, platelets become activated, undergo pronounced morphological changes, and release granule contents, including adenosine diphosphate (ADP) and thromboxane A2 (TXA2). In activated platelets, glycoprotein GP IIb/IIIa undergoes conformational changes that markedly increase its binding affinity. By binding to plasma fibrinogen, GP IIb/IIIa mediates interplatelet bridging and subsequently triggers platelet aggregation. Continuous release of these agonists further promotes platelet recruitment and activation, progressively amplifying the response and ultimately forming a relatively loose platelet plug at the injury site to achieve initial hemostatic occlusion [11,12,13].

During primary hemostasis, the platelet plug not only provides temporary vascular sealing but also supplies a phospholipid membrane surface that serves as a crucial catalytic interface for the coagulation cascade in secondary hemostasis. The extrinsic pathway is initiated through the formation of the tissue factor-VIIa complex (TF-VIIa) between TF released from injured tissues and circulating factor VIIa, which directly activates factors X and IX and functions as the rapid initiation signal of the coagulation process [14,15]. In contrast, the intrinsic pathway is initiated when factor XII comes into contact with negatively charged surfaces exposed at the injury site, such as collagen, followed by the sequential activation of factors XI and IX [16]. This pathway plays an essential role in the amplification and stabilization of coagulation responses. Ultimately, both pathways converge into the common coagulation pathway, in which activated factor X (FXa), together with cofactor Va, Ca^2+^, and the platelet phospholipid membrane, assembles into the prothrombinase complex that catalyzes the conversion of prothrombin into thrombin (factor IIa). Thrombin plays a central role throughout this process. It converts fibrinogen into fibrin monomers, which subsequently polymerize into a fibrin network, while simultaneously activating platelets and multiple coagulation cofactors, including factors V, VIII, and XI, thereby further amplifying the coagulation response. In addition, thrombin activates factor XIII, promotes fibrin crosslinking, and enhances the mechanical stability of the blood clot [15,17].

Ultimately, the interwoven fibrin network tightly encapsulates the platelet plug and erythrocytes, forming a stable red thrombus capable of achieving sustained hemostasis. Throughout this process, platelets provide the essential catalytic interface for coagulation reactions, whereas thrombin further promotes platelet activation, demonstrating the highly coordinated interplay between primary and secondary hemostasis [18]. Physiological hemostasis is inherently a dynamic equilibrium process. As coagulation progresses, the fibrinolytic system is subsequently activated to limit excessive thrombus formation and prevent pathological coagulation. Therefore, the design of ideal hemostatic materials should not only enhance coagulation efficiency but also preserve this physiological balance, ensuring that the materials do not interfere with subsequent tissue repair or thrombus degradation after fulfilling their hemostatic function [7].

### 2.2. Mechanisms of Action of Hemostatic Hydrogels

The design of modern hemostatic hydrogels is no longer restricted to a single mechanism; instead, it integrates physical, chemical, and biological strategies to establish synergistic hemostatic systems. In general, the mechanisms underlying the hemostatic effects of hydrogels can be categorized into the following three aspects.

Owing to their excellent hydrophilicity and porous network structures, hydrogels can rapidly absorb plasma and interstitial fluid upon contact with the wound surface. This process leads to the local concentration of platelets, erythrocytes, and coagulation factors, thereby increasing the local concentration of coagulation substrates and promoting activation of the natural coagulation cascade. Simultaneously, the volumetric expansion of hydrogels after fluid absorption exerts sustained yet relatively mild physical compression on the wound site, slowing local blood flow and providing a stable microenvironment for clot formation [19,20]. For instance, a dual-network hydrogel constructed from Bletilla striata polysaccharide and gelatin, exhibiting a swelling ratio exceeding 500% and high porosity, was reported to significantly shorten whole-blood clotting time in vitro [21]. This mechanism mainly promotes coagulation through physical effects and generally exhibits favorable biosafety.

Beyond blood concentration and fluid absorption, hydrogels can actively participate in coagulation initiation through charge-mediated regulation. The incorporation of negatively charged nanoparticles, such as kaolin or silicate-based materials, as well as negatively charged biopolymers, including alginate, can mimic the negatively charged interfaces exposed at injury sites, thereby specifically activating coagulation factor XII and initiating the intrinsic coagulation pathway, ultimately accelerating thrombin generation [22,23,24]. In contrast, hydrogels enriched with positively charged groups, particularly amino-containing materials such as chitosan and its derivatives, can strongly interact with negatively charged platelets and erythrocytes through electrostatic interactions. This promotes nonspecific cell aggregation, platelet pseudopod extension, and platelet thrombus formation, even under conditions of insufficient coagulation factors [25]. In addition to charge-mediated coagulation activation, hydrogels can also function as carriers for the controlled delivery of bioactive molecules, including thrombin and tranexamic acid [26,27]. Furthermore, the grafting of arginine-glycine-aspartic acid (RGD) peptides or other self-assembling peptides enables specific platelet recognition and regulation, thereby further enhancing coagulation efficiency [28,29]. In addition to coagulation regulation, hydrogels can function as physical barriers by providing immediate mechanical sealing through strong wet adhesion on moist tissue surfaces. In particular, hydrogel systems possessing in situ gelation capability can effectively penetrate and fill irregular wound cavities [30]. Such materials remain effective even under conditions of impaired coagulation function [20,31]. Their adhesive strategies are commonly based on biomimetic chemistry, including mussel-inspired catechol groups and reactive ester structures such as N-hydroxysuccinimide (NHS) esters, which can form stable covalent or noncovalent interactions with amino and thiol groups on tissue surfaces [6,32,33,34]. To overcome the detrimental effects of the interfacial hydration layer on wet adhesion in blood-rich environments, one widely adopted strategy involves the introduction of hydrophobic groups to displace interfacial water molecules, thereby promoting intimate contact between the material and tissue surfaces [35]. Another strategy utilizes blood itself to construct the interfacial adhesive structure. Based on the blood impregnation mechanism, microparticles absorb water, aggregate, and undergo in situ crosslinking, thereby reducing competitive occupation of adhesion sites by interfacial fluids and forming a stable composite blood clot capable of achieving rapid hemostasis [36].

Overall, the hemostatic effects of hydrogels are primarily achieved through blood concentration and physical compression, charge-mediated coagulation activation, and adhesion-based mechanical sealing. Contemporary hydrogel design increasingly integrates multiple hemostatic mechanisms to achieve more rapid, efficient, and stable hemostatic performance. This trend reflects the ongoing transition of hemostatic hydrogels from passive physical coverage toward active and multifunctional regulation, thereby providing broader opportunities for future functional expansion and clinical applications.

## 3. Material Design of Natural Polymer-Based Hemostatic Hydrogels

Owing to their excellent biocompatibility, intrinsic bioactivity, biodegradability, and ease of processing, natural polymer-based hemostatic materials have attracted extensive attention in the biomedical field. At present, the most widely investigated natural materials for hemostatic hydrogel construction mainly include polysaccharides and proteins. The following sections summarize recent advances in natural hemostatic materials. Rather than simply categorizing materials according to their types, this chapter focuses on how different molecular modification strategies address the inherent performance limitations of prototype systems and existing commercial products to improve overall material performance. Table 1 summarizes the material sources, hemostatic mechanisms, major advantages and disadvantages, and representative molecular modification strategies associated with polysaccharide- and protein-based hemostatic hydrogels.

### 3.1. Polysaccharides

Polysaccharide-based materials are abundant in nature, highly structurally tunable, and possess intrinsic hemostatic activity and favorable biological properties, making them ideal scaffold materials for constructing high-performance hemostatic systems. Although different polysaccharides exhibit distinct molecular backbones—for example, chitosan contains an N-acetylglucosamine-based backbone, whereas alginate is primarily composed of glucuronic acid units—the molecular engineering strategies employed for their hemostatic applications exhibit considerable commonality. In general, targeted chemical modifications are introduced onto reactive side-chain functional groups, including hydroxyl (–OH), amino (–NH_2_), and carboxyl (–COOH) groups, through approaches such as quaternization, hydrophobic alkylation, and oxidative modification. These strategies can regulate charge density, hydrophilic–hydrophobic balance, interfacial interactions, and network dynamics, thereby enhancing the solubility, wet adhesion, mechanical adaptability, and biological functionality of polysaccharide-based hemostatic materials under physiological conditions.

This section focuses on representative polysaccharides and discusses how molecular design strategies can regulate their physicochemical properties and biological activities through chemical modification, ultimately enhancing their performance in hemostatic applications.

#### 3.1.1. Chitosan

Chitosan (CS), derived primarily from the exoskeletons of crustaceans, is the only naturally occurring cationic polysaccharide identified in nature [37]. It possesses excellent biodegradability [38], biocompatibility [39], intrinsic hemostatic and antithrombotic activities [40], as well as broad-spectrum antibacterial properties [41,42]. The hemostatic activity of chitosan mainly originates from its protonated amino groups (–NH_3_^+^), which can electrostatically attract negatively charged erythrocytes and platelets, thereby inducing cellular aggregation and promoting platelet activation [43,44,45]. Commercial hemostatic products based on chitosan, such as HemCon^®^ and Celox^®^, have already achieved clinical application [46]. However, native chitosan still suffers from several intrinsic limitations, including poor solubility under physiological pH conditions, insufficient mechanical stability, and excessive gelation after water absorption [47,48,49]. These shortcomings substantially restrict its clinical performance and broader translational potential. The amino groups at the C2 position (C2–NH_2_) and hydroxyl groups at the C6 position (C6–OH) along the chitosan molecular backbone serve as the principal reactive sites for chemical modification. Accordingly, molecular engineering strategies aimed at regulating solubility and charge density have become common approaches for optimizing chitosan-based hemostatic hydrogels. Jiang et al. introduced hydrophilic carboxyl groups through carboxymethylation, thereby broadening the pH-dependent solubility range of chitosan and endowing the material with the ability to promote burn wound epithelialization [50]. Quaternization represents another classic and highly effective modification strategy. Representative derivatives, including N-[(2-hydroxy-3-trimethylammonium)propyl] chitosan chloride (HTCC) and N,N,N-trimethyl chitosan (TMC), not only markedly improve the aqueous solubility of chitosan but also enhance electrostatic interactions with erythrocytes and negatively charged bacterial membranes through their high-density positive charges, thereby simultaneously achieving efficient hemostasis and broad-spectrum antibacterial activity [51].

Recent studies have further advanced the understanding of the charge-mediated hemostatic mechanism of chitosan. Huang et al. demonstrated that the –NH_3_^+^ groups on protonated chitosan (PCS) chains and their derivatives not only mediate electrostatic adsorption but also function as critical active sites for coagulation factor recruitment, plasma protein assembly, and acceleration of the coagulation cascade [52]. Nevertheless, excessively high local charge density may induce hemolysis or abnormal platelet activation, thereby raising biosafety concerns. To address the cytotoxicity associated with highly cationic quaternized chitosan derivatives such as HTCC, Liu et al. proposed a charge-balancing strategy by introducing a zwitterionic copolymer poly([2-(methacryloyloxy)ethyl]trimethylammonium chloride-co-acrylic acid) (pMATC-co-AA) to form an electrostatic complex with HTCC. This strategy significantly improved biocompatibility while also enabling on-demand material removal [25]. Therefore, future research on chitosan-based hemostatic hydrogels should continue to focus on achieving an optimal balance between coagulation efficiency and biosafety.

**Figure 3:**
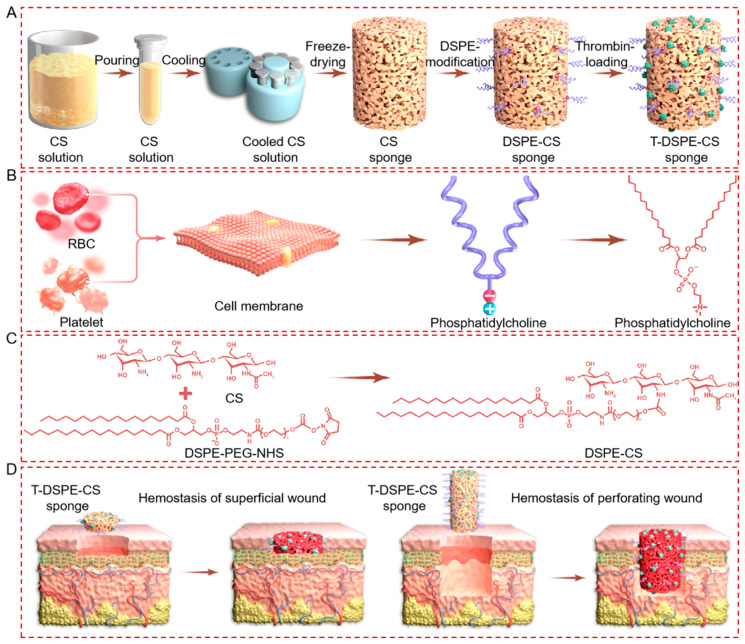
Preparation and hemostatic applications of the thrombin-loaded DSPE-CS (T-DSPE-CS) sponge. (**A**) The preparation process of the T-DSPE-CS sponge. (**B**) The concept and (**C**) mechanism of DSPE-modification. (**D**) The hemostatic application of the T-DSPE-CS sponge in managing uncontrolled coagulopathic hemorrhage from superficial and perforating wounds. Reprinted with permission from ref. [55]. Copyright 2024 Du et al. Published by Elsevier Ltd.

The introduction of hydrophobic side chains, including alkyl or aromatic groups, can generate localized hydrophobic domains within the molecular network, thereby reducing the interference of interfacial water and enhancing wet adhesion performance. Simultaneously, hydrophobic interactions contribute to improved mechanical stability of the material. N-alkylation modification can further endow chitosan molecules with amphiphilic characteristics [53,54]. To address the hemostatic demands under coagulopathy conditions, Du et al. developed a multifunctional hemostatic sponge integrating both active and passive hemostatic pathways. First, an intrinsically elastic chitosan sponge was fabricated via a freeze-drying process (Figure 3A). Subsequently, alkyl chains and phosphate groups were covalently anchored onto the sponge surface through amidation reactions between the amino groups of CS and the N-hydroxysuccinimide (NHS) ester groups of DSPE-PEG-NHS (Figure 3A–C). The authors proposed that these grafted functional groups could interact with blood cells through hydrophobic interactions and electrostatic interactions (Figure 3B), thereby promoting blood cell adhesion, aggregation, and activation and ultimately accelerating the coagulation process. Finally, thrombin was physically loaded into the chemically modified sponge, termed DSPE-CS, endowing the material with the ability to activate the endogenous coagulation cascade. In heparinized rat models of tail amputation, liver surface injury, and liver perforation, the sponge effectively controlled uncontrolled coagulopathic bleeding induced by both superficial and perforating wounds (Figure 3D) [55]. In addition, the incorporation of polyphenolic molecules can further enhance the wet adhesion performance of chitosan-based hydrogels. Tannic acid (TA), which is rich in catechol and galloyl groups, possesses structural features analogous to the 3, 4-dihydroxy-L-phenylalanine (DOPA) motifs found in mussel foot proteins and is therefore widely employed in the construction of wet-adhesive hemostatic hydrogels. Guo et al. fabricated a dynamic hydrogel network based on TA and quaternized chitosan (QCS). Benefiting from the excellent reactive oxygen species (ROS)-scavenging capability of TA, the resulting hydrogel exhibited both rapid hemostatic performance and anti-inflammatory wound-healing activity [56]. Similarly, the TA-mediated gel system developed by Zhang et al. demonstrated excellent wet tissue adhesion, providing new insights into the molecular design of bioadhesive hemostatic materials [30].

#### 3.1.2. Cellulose

Cellulose is a linear polysaccharide composed of D-glucopyranose units linked through β-1, 4-glycosidic bonds. As the most abundant and widely distributed natural polysaccharide, cellulose accounts for more than 50% of the total carbon content in the plant kingdom [57]. The dense hydrogen-bonding network formed between cellulose molecular chains confers exceptionally high crystallinity and chemical stability. While this highly ordered structure provides essential mechanical strength for plants, it also represents a major obstacle for biomedical hemostatic applications. Owing to the absence of endogenous cellulolytic enzymes in the human body, together with the poor solubility and absorbability of native cellulose, direct implantation of unmodified cellulose can readily induce foreign body granulomatous reactions [58]. To improve biocompatibility and degradability, oxidative modification has been extensively employed to convert cellulose into absorbable hemostatic materials, exemplified by commercially available oxidized regenerated cellulose (ORC) products such as Surgicel^®^ [59,60]. Wang et al. developed a sprayable hydrogel system based on TEMPO-mediated oxidized cellulose nanofibers (TOCNs), which exhibited excellent biocompatibility and hemostatic performance [61]. Zhang et al. further combined TOCNs with chitosan nanofibers (CSNFs) through crosslinking to fabricate a nanofibrous hemostatic sponge suitable for non-compressible hemorrhage applications [62]. Oxidative treatment not only disrupts the crystalline structure of cellulose, thereby improving biodegradability, but also endows the material with a characteristic acid-mediated hemostatic mechanism. Specifically, the carboxyl groups introduced into the molecular chains of ORC lower the local pH at the wound site. Under acidic conditions, hemoglobin releases Fe^3+^ ions, which can interact with cellulose chains to enhance blood clot stability while simultaneously promoting platelet aggregation and inhibiting bacterial proliferation [59].

However, excessively acidic microenvironments may also produce adverse biological effects. Under fully hydrated conditions, the local pH surrounding ORC materials can decrease to approximately 1.7. Such strong acidity may induce severe tissue inflammation, damage surrounding nerves, and even inhibit thrombin activity [63]. To address these limitations, multifunctional composite modification strategies have become an important research direction. For example, Alipour et al. developed a multifunctional regenerated cellulose gauze (PD-OCMRC) through a four-step chemical modification process involving carboxymethylation, coordination of calcium and zinc ions with carboxyl groups, oxidation, and dopamine self-polymerization. This material effectively activated the coagulation cascade and promoted fibrin network formation while simultaneously exhibiting strong wet adhesion and antibacterial activity. Its hemostatic efficacy was validated in rat and rabbit liver hemorrhage models, where it significantly outperformed commercial products such as ChitoCell^®^ and BloodStop^®^ [64]. In addition, carboxymethyl cellulose (CMC) intrinsically forms a stable anionic polyelectrolyte structure, providing an ideal platform for ionic regulation. Yao et al. prepared calcium-loaded carboxymethyl cellulose powder (Ca-ex-CMC) using a modified ion-exchange strategy, thereby endowing the material with excellent fluid absorption capability. Upon contact with blood or other fluids, the material rapidly absorbed moisture and formed a gel, while simultaneously releasing Ca^2+^ ions that functioned as coagulation factor IV to promote fibrin formation [65]. Such strategies no longer rely solely on acidic microenvironments to induce coagulation; instead, they regulate the coagulation process through ionic interactions, integrating biochemical procoagulant effects with physical sealing mechanisms. Nevertheless, minimizing inflammatory responses and enzymatic interference caused by acidic microenvironments remains a major challenge for the future clinical translation of cellulose-based hemostatic hydrogels.

#### 3.1.3. Alginate

Alginate is primarily derived from brown algae and certain bacterial species. It is a linear anionic polysaccharide composed of β-D-mannuronic acid (M units) and α-L-guluronic acid (G units). The G blocks within the alginate molecular structure can rapidly undergo ionic gelation through coordination with multivalent cations, most commonly Ca^2+^ ions. In addition, the released Ca^2+^ can function as coagulation factor IV and participate in activation of the coagulation cascade, thereby promoting hemostasis [66]. However, the ionically crosslinked network formed through such coordination interactions exhibits limited stability under physiological conditions and is susceptible to erosion by body fluids, making it difficult to maintain long-term structural integrity [37,67]. To overcome these intrinsic limitations, current molecular engineering strategies for alginate-based hemostatic hydrogels mainly focus on introducing more stable covalent crosslinking networks or enhancing structural stability through hybridization with other bioactive components. For example, Zhao et al. developed a self-healing oxidized dextran/sodium alginate (SODex) hydrogel dressing. In this design, dynamic covalent bonds were introduced into the ionically crosslinked network through Schiff base reactions and hydrogen-bonding interactions, significantly improving both adhesion strength and network cohesion. As a result, the hydrogel exhibited enhanced hemostatic efficacy in mouse liver and tail hemorrhage models while simultaneously accelerating burn wound healing through promotion of epithelial regeneration and angiogenesis [68]. Beyond covalent crosslinking strategies, the incorporation of inorganic nanomaterials has also attracted considerable attention. Zhu and colleagues designed a composite dressing consisting of ultralong hydroxyapatite (HAP) nanowires and calcium alginate. In this system, alginate functioned as a flexible matrix encapsulating the nanowire framework, whereas the HAP nanowires served as reinforcing components to enhance the overall mechanical properties of the material. Simultaneously, the sustained presence of calcium ions within the network promoted microvascularization and epithelial cell proliferation, thereby reducing scar formation during wound healing [69]. In terms of ionic regulation, recent studies have further introduced metal ions other than Ca^2+^ to construct multicomponent coordination networks. For instance, Abhishek Kumar et al. incorporated Zn^2+^ into an alginate-calcium system, thereby enhancing network stability through synergistic multivalent ionic coordination. In a BALB/c mouse tail amputation model, the resulting material reduced hemostasis time by approximately 60%, exhibiting performance comparable to that of the commercially available Celox^®^ hemostatic granules and demonstrating considerable translational potential [70]. Although these modification strategies significantly improve the mechanical properties and erosion resistance of alginate under physiological conditions, the design of alginate-based hemostatic materials still requires a careful balance between structural stability and degradation behavior. Moreover, the long-term performance and biosafety of these materials in complex hemorrhagic environments remain to be systematically evaluated.

#### 3.1.4. Hyaluronic Acid

Unlike alginate, which primarily relies on ionic crosslinking mediated by G units within its molecular backbone, hyaluronic acid (HA), as a major component of the ECM, is more specifically designed to mimic and regulate cellular behaviors. Hyaluronic acid is composed of repeating disaccharide units of β-D-glucuronic acid and N-acetyl-β-D-glucosamine. It is a linear, anionic, non-sulfated polysaccharide [71] and also a key component of the ECM. Owing to its excellent biocompatibility and its specific interactions with cell-surface receptors such as CD44, the function of HA in hemostatic hydrogels is primarily associated with the regulation of the biointerface and wound microenvironment. However, native HA exhibits strong hydrophilicity and lubricating properties, making it difficult to establish stable adhesion at wet bleeding interfaces. In addition, HA lacks the intrinsic capability to directly initiate the coagulation cascade. To overcome the insufficient wet adhesion of HA-based materials, a common molecular modification strategy involves introducing functional groups capable of forming dynamic covalent interactions with cell membrane glycoproteins, thereby constructing stable interfacial binding sites. Li et al. reported a single-component HA-based hydrogel system in which phenylboronic acid (PBA) groups were grafted onto the HA backbone to construct an HA-3APBA hydrogel. In this system, PBA not only participated in network formation through dynamic boronate ester bonds, thereby endowing the hydrogel with self-healing and injectability, but also formed specific dynamic complexes with cis-diol structures on the cell membrane surface, which significantly enhanced tissue adhesion. This multifunctional molecular recognition mechanism enabled the hydrogel to exhibit relatively stable hemostatic performance in a mouse tail-amputation model [72]. Building upon the enhancement of wet interfacial adhesion, subsequent molecular engineering strategies further focused on imparting HA with bioactivity capable of actively regulating the coagulation cascade. Liu et al. grafted adenosine diphosphate (ADP) onto HA molecular chains through an amidation reaction. ADP functions as a ligand for platelet P2Y1 and P2Y12 receptors, thereby inducing platelet activation and platelet aggregation. Following this modification, HA no longer served merely as a physical barrier or spatial-filling material, but also participated in platelet recruitment and activation processes, thereby modulating the coagulation process to a certain extent. Such molecular designs based on the introduction of bioactive signaling molecules provide new insights into the development of hemostatic hydrogels with active regulatory capabilities [73].

#### 3.1.5. Starch

**Figure 4:**
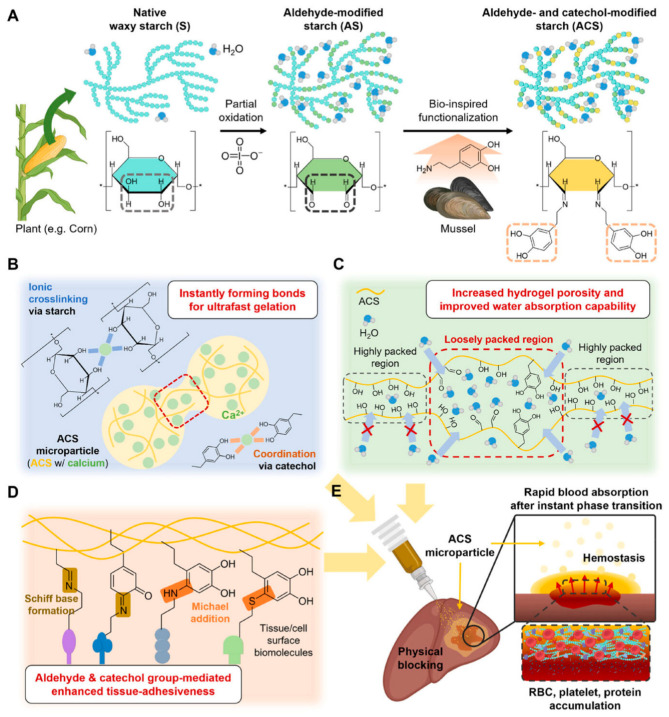
Synthesis of aldehyde- and catechol-modified starch (ACS) polymer and characteristics of ACS microparticle (MP). (**A**) Schematic illustration of the synthesis procedures of the ACS polymer and water accessibility of the starch polymer in each stage. (**B**) Calcium-mediated, instantly forming bonds among the ACS polymers in the ACS-MP. (**C**) Enhanced water accessibility of the ACS polymer and improved water absorption capability driven by chemical modifications. (**D**) Chemical bonding of the aldehyde group and catechol group in the ACS polymer with several functional groups in biomolecules for robust bio-adhesive properties of the ACS-MP. (**E**) Plausible mechanisms of synergistically enhanced hemostatic capability of the ACS-MP. Asterisks (*) denote the continuation of the polymer backbone. Reprinted with permission from ref. [79]. Copyright 2025 An et al. Advanced Science is published by Wiley-VCH GmbH.

Natural starch, owing to the abundant hydrophilic hydroxyl groups within its molecular backbone, exhibits excellent fluid absorption capacity, swelling behavior, and biocompatibility. It has been widely applied in FDA-approved hemostatic powders, such as Arista^®^ and PerClot^®^ [74,75]. However, from the perspective of molecular bioactivity, starch-based materials remain biologically inert polysaccharides. Their molecular structures lack endogenous charges and specific biorecognition motifs, and they are susceptible to rapid enzymatic degradation in vivo due to the action of amylases. Therefore, compared with cationic polysaccharides such as chitosan or protein-based materials containing integrin-binding motifs, the central objective of starch molecular engineering is to transform starch from an inert material into a functional system capable of actively regulating hemostasis. Given the electrically neutral nature of native starch, quaternization is commonly employed to introduce positive charges into the molecular chains. For instance, modification with 3-chloro-2-hydroxypropyl trimethylammonium chloride (CHPTAC) can generate a high density of positively charged sites along the starch backbone, thereby altering its interactions with blood components and endowing the material with the capability to adsorb erythrocytes and platelets [76]. Liu et al. further proposed a synergistic molecular modification strategy combining carboxymethylation and methacrylation, in which carboxymethyl groups enhanced the hydrophilicity and charge density of the molecular chains, while methacrylate groups introduced polymerizable double bonds for the construction of stable covalent crosslinking networks [77]. Furthermore, to improve bioactivity, Wu et al. grafted serotonin onto the starch backbone to construct a functionalized starch material, enabling the material to participate in platelet activation and thereby promote initiation of the coagulation process [78]. With respect to wet adhesion and mechanical adaptability, current starch modification strategies have mainly focused on the construction of biomimetic interfaces and dynamic networks to overcome the intrinsic limitations of native starch. An et al. designed a highly absorbent and self-gelling microparticle (MP) system based on the aldehyde- and catechol-modified starch (ACS). Specifically, corn amylopectin was partially oxidized to introduce aldehyde groups (Figure 4A). Subsequently, dopamine (DA) was conjugated to incorporate mussel-inspired catechol groups, while Ca^2+^ ions were intercalated to enable ionic crosslinking-mediated ultrafast wet-state gelation (Figure 4B). These modifications disrupted intermolecular hydrogen bonding, thereby enhancing water absorption capacity and increasing hydrogel porosity (Figure 4C). In addition, aldehyde and catechol groups synergistically promoted covalent tissue adhesion through Schiff base reactions and Michael addition reactions (Figure 4D). When applied in the form of microparticles, ACS-MPs rapidly absorbed blood and transformed into an adhesive hydrogel, thereby physically sealing irregular wounds while simultaneously concentrating blood cells and coagulation factors at the wound site (Figure 4E). Effective hemostatic performance was further confirmed in both mouse and porcine liver hemorrhage models [79]. In addition, by utilizing the ortho-diol structures present on glucose units, reversible boronate ester bond systems (St-SP) or dynamic covalent networks based on oxidized starch can also be constructed [80,81]. These dynamic multicrosslinking strategies effectively improve the brittleness of conventional starch hydrogels and enable the materials to better adapt to irregular wound environments.

From the perspective of the synergistic relationship among molecular structure, interfacial behavior, and macroscopic performance, although the chemical forms of polysaccharide molecular modification strategies are highly diverse, their underlying mechanisms exhibit substantial consistency. Specifically, these strategies regulate interfacial interactions within the blood environment through modulation of charge distribution, hydrophobic interactions, and dynamic covalent bonds, thereby ultimately determining the hemostatic performance of the materials. Nevertheless, the effectiveness of such systems in complex hemorrhagic environments still strongly depends on the stability and persistence of these interfacial interactions.

### 3.2. Proteins

Unlike polysaccharide-based systems, which primarily rely on physicochemical interactions, the functionality of protein-based materials is more strongly associated with their specific higher-order structures and biorecognition motifs.

#### 3.2.1. Collagen and Gelatin

Collagen is the most abundant and widely distributed structural protein in animals [82]. Current research and development of collagen-based hemostatic materials mainly focus on native collagen and its denatured derivative, gelatin. Collagen molecules possess a characteristic triple-helix structure and contain arginine–glycine–aspartic acid (RGD) sequences as well as GFOGER motifs. These structural domains can rapidly induce platelet adhesion, platelet activation, and platelet aggregation during the early stage of injury, thereby initiating the endogenous coagulation cascade [83,84]. In contrast, gelatin is a denatured product generated from collagen under high-temperature conditions. Although gelatin partially loses its higher-order structure, it retains RGD sequences and matrix metalloproteinase (MMP)-sensitive cleavage sites. Moreover, its molecular chains contain abundant exposed amino groups, carboxyl groups, and hydroxyl groups, making it an ideal platform for molecular modification [85]. However, collagen- or gelatin-based gels that mainly rely on physical entanglement and hydrogen bonding for structural stabilization are highly sensitive to temperature and tend to become structurally loose or even disintegrate under physiological conditions (37 °C). In addition, the native molecular chains lack sufficient wet adhesion to resist arterial hemorrhage, and they undergo rapid enzymatic degradation within enzyme-rich wound environments, making it difficult to maintain a long-lasting physical barrier [83,86]. To overcome the insufficient mechanical strength of gelatin at physiological temperature, amino groups on gelatin side chains are frequently modified with methacrylic anhydride to introduce methacrylate groups, thereby producing photocrosslinkable gelatin methacryloyl (GelMA). This molecular modification enables GelMA to undergo free-radical polymerization under photoinitiation, forming a covalent crosslinking network and transforming the original reversible physical gel into a structurally stable hydrogel. By regulating the degree of substitution and photopolymerization conditions, the mechanical properties and swelling behavior of GelMA hydrogels can be precisely controlled to satisfy the requirements of applications such as hemostatic barriers [87]. Building upon this strategy, several studies have further developed asymmetric bilayer structures based on GelMA to achieve selective adhesion to wound tissues while minimizing nonspecific adhesion to surrounding tissues. For example, by introducing N-acetylcysteine (NAC) into gelatin molecular chains and utilizing sulfhydryl groups on the side chains to participate in disulfide exchange reactions, the material can form stable covalent interactions with tissue proteins, achieving an adhesion strength of approximately 1.35 kPa under wet conditions. Meanwhile, the opposite side retains a photocrosslinked network structure to reduce the risk of postoperative tissue adhesion, thereby enabling region-selective hemostasis and adhesion [88].

Natural peptide chains generally lack reactive groups capable of penetrating the interfacial hydration layer of blood-covered tissues, resulting in insufficient wet adhesion. Inspired by both fibrin polymerization and mussel adhesion mechanisms, researchers developed a gelatin-DOPA knob/fibrinogen (GDK/Fg) hydrogel system. In this design, gelatin molecular chains were simultaneously modified with catechol groups (DOPA) and specific knob peptide motifs. These peptide motifs interacted with Fg through knob–hole recognition interactions, while the catechol groups enhanced cohesive properties through multiple noncovalent interactions, collectively promoting the formation of the GDK/Fg hydrogel network. The hydrogel further interacted with tissue surfaces and Fg through catechol-mediated interfacial interactions and knob peptides, thereby significantly enhancing wet interfacial adhesion [89]. In addition, utilizing the chemical reactivity of polysaccharides to achieve mild protein modification represents another effective strategy. Studies have demonstrated that epoxy-activated Bletilla striata polysaccharide (EBSP) can undergo ring-opening crosslinking reactions with amino groups on collagen side chains. This process increases the crosslinking density while preserving the integrity of the collagen triple-helix structure, enabling the two components to exert synergistic effects during hemostasis [90]. Overall, protein-based hemostatic hydrogels exhibit a pronounced trade-off between mechanical reinforcement and preservation of bioactivity. Although excessive crosslinking can enhance structural stability, it may simultaneously shield critical biorecognition motifs and reduce cellular responsiveness. Moreover, under enzyme-rich wound conditions, these materials still suffer from rapid enzymatic degradation, making it challenging to maintain long-term structural stability in complex hemorrhagic environments, particularly under severe bleeding conditions. Further optimization is therefore still required.

#### 3.2.2. Silk Fibroin

**Figure 5:**
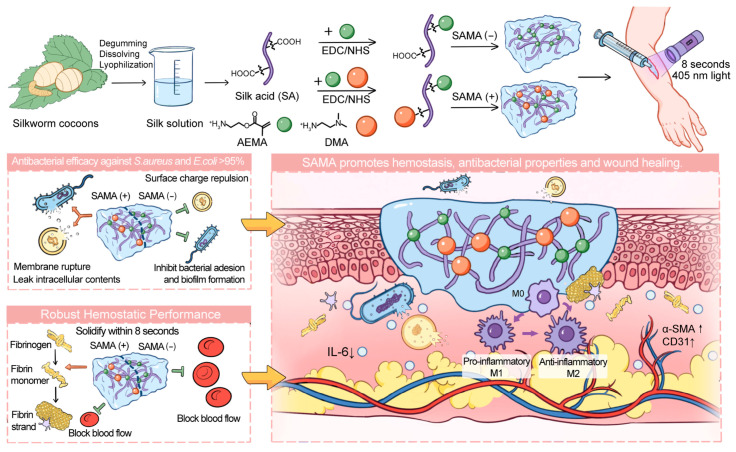
Schematic illustration of the design, multifunctional properties, and wound healing mechanism of the charged silk-based hydrogel (SAMA) hydrogels. Derived from natural silk fibroin, the hydrogel is functionalized with Aminoethyl methacrylate (AEMA) and dimethylamino (DMA) to enable rapid photocrosslinking and a positive surface charge. Upon 405 nm light exposure, SAMA(+) rapidly gels in situ and achieves ultrafast hemostasis by forming a dense interfacial seal and promoting platelet adhesion, activation (CD62P, PAC-1), and fibrin network formation through electrostatic interactions. The hydrogel exhibits enhanced antibacterial activity (>95% against *S. aureus* and *E. coli*), robust hemostasis, and immune modulation. It promotes M2 macrophage polarization, angiogenesis, and tissue regeneration, offering an integrated solution for infected and bleeding wounds. Upward arrows (↑) indicate upregulation or increase, and downward arrows (↓) indicate downregulation or decrease. Reprinted with permission from ref. [96]. Copyright 2025 Wang et al. Publishing services by Elsevier B. V. on behalf of KeAi Communications Co. Ltd.

Silk fibroin (SF), derived from the domestic silkworm, is a natural macromolecular protein composed of a light chain with a molecular weight of approximately 26 kDa and a heavy chain of approximately 390 kDa connected through disulfide bonds. Its amino acid sequence is enriched with repetitive Gly–Ala–Gly–Ala–Gly–Ser motifs, which can spontaneously self-assemble into β-sheet structures. These higher-order structures endow the material with excellent mechanical strength and enable SF to exhibit more stable and controllable degradation behavior than many other natural biopolymers under both in vitro and in vivo conditions [91,92]. Nevertheless, SF hydrogels mainly rely on β-sheet-mediated physical crosslinking for gelation, and this self-assembly process generally requires more than 10 min, making it difficult to satisfy the rapid-response requirements for acute massive hemorrhage management [93]. In addition, SF molecular chains lack functional groups capable of rapidly establishing stable interfacial interactions with wet tissues. Under conditions involving arterial hemorrhage and continuous blood flow erosion, the interfacial adhesion of SF-based materials remains insufficient, thereby limiting their ability to achieve effective wound sealing [94]. Similar to collagen-based systems, methacrylation is also a commonly employed molecular modification strategy for SF. However, the purpose of this modification differs substantially between the two materials. In gelatin systems, methacrylation is primarily utilized to improve structural stability under physiological conditions, whereas in SF systems, it is mainly introduced to overcome the inherently slow gelation kinetics associated with molecular self-assembly. By incorporating photoresponsive methacrylate (MA) groups into SF molecular chains, the gelation process can be triggered by external light stimulation, thereby enabling rapid photocuring [95]. Building upon this strategy, to further integrate coagulation-regulating functionality, another study developed a chemically engineered charged silk fibroin-based hydrogel (SAMA) that combines ultrafast gelation, intrinsic antibacterial activity, and programmable bioactivity. The SAMA hydrogel was fabricated through dual side-chain modification of silk fibroin (SF). Specifically, 2-aminoethyl methacrylate (AEMA) introduced methacrylate groups to enable rapid visible-light-induced photocrosslinking, whereas dimethylamino (DMA) groups endowed the hydrogel with a moderate positive charge characterized by relatively low surface charge density and favorable biocompatibility. This molecular-level engineering strategy simultaneously regulated the photocrosslinking kinetics and interfacial charge distribution within a single SF network. The resulting SAMA hydrogel exhibited ultrafast gelation (<8 s), tunable surface charge properties, and broad-spectrum antibacterial activity. Its positively charged network further enhanced both hemostatic and antibacterial performance, while the hierarchical structure facilitated cell migration, angiogenesis, and macrophage polarization toward the M2 phenotype. In vivo studies demonstrated that SAMA hydrogels significantly accelerated wound closure and promoted tissue regeneration, highlighting their considerable potential as next-generation bioactive wound dressings for emergency applications (Figure 5) [96]. However, although multiple chemical modification strategies can significantly improve material functionality, they may also increase system complexity and introduce potential biosafety concerns, which still require comprehensive evaluation. In addition to structural reinforcement and interfacial regulation, silk fibroin itself may participate in oxidative microenvironment modulation. Previous studies demonstrated that SF could effectively scavenge hydroxyl radicals generated from long-term stored water-soluble fullerene systems while simultaneously reducing oxidative damage to endothelial cells. This antioxidant activity was associated with accelerated fullerene degradation and suppression of reactive oxygen species accumulation, suggesting that SF-based biomaterials may contribute not only to hemostasis but also to the protection of vascular and tissue microenvironments under oxidative stress conditions [97].

To improve wet interfacial adhesion, Wang et al. combined SF with TA to construct a hydrogel network with strong tissue adhesive capability and further demonstrated that acidic environments could markedly enhance its adhesive performance. The study revealed that, under gastric acid conditions, elevated proton concentrations weakened electrostatic shielding effects at the interface while simultaneously altering the arrangement of interfacial water molecules. This process strengthened both electrostatic interactions and van der Waals interactions between the SF–TA composite and keratin within the gastric mucosal epithelium, resulting in an increase in adhesion strength of more than 50% compared with that under physiological pH conditions. Moreover, the stable β-sheet structures within SF promoted strong intermolecular interactions with TA, enabling the material to maintain structural stability within the gastric fluid environment for more than 5 days. Further incorporation of a fibrin-promoting SF nanofiber/protein crown composite (SFNPC) provided sustained interfacial protection, reduced the risk of rebleeding, and promoted gastric mucosal repair within 5 days [98].

#### 3.2.3. Fibrin

Fibrin is a class of natural biopolymers derived from the blood system. Owing to its excellent biocompatibility and in situ gelation capability, fibrin gels (FGs) have been widely utilized in surgical applications. Their hemostatic mechanism primarily relies on mixing fibrinogen and thrombin solutions to generate fibrin clots, corresponding to the terminal stage of the coagulation cascade [99]. In addition to hemostatic functionality, the natural three-dimensional network structure of fibrin also serves as an ECM-like scaffold, promoting cell migration and angiogenesis through RGD motifs [100]. Currently, commercially available allogeneic fibrin sealants, such as Tisseel^®^ and Evicel^®^, have been widely applied in clinical practice [101]. However, native fibrin gels exhibit insufficient mechanical strength and are highly susceptible to rapid degradation by the fibrinolytic system. Consequently, they are unable to independently provide stable sealing under conditions of high-pressure arterial hemorrhage, which has become a major focus of subsequent molecular engineering strategies [102]. To address the insufficient wet adhesion of native fibrin systems, which mainly rely on noncovalent knob–hole interactions and limited factor XIIIa-mediated enzymatic crosslinking, sequential crosslinking strategies have gradually attracted increasing attention. Yu et al. proposed a dual-network structural construction strategy in which the rapid enzymatic reaction between fibrinogen and thrombin (approximately 1.3 s) was first utilized to establish an initial physical barrier at the wound site while simultaneously confining the photosensitive precursor gelatin methacryloyl (GelMA) within the fibrin network. Subsequently, a second dense covalent crosslinking network was generated through photoinitiated free-radical polymerization. This multinetwork structural design significantly enhanced the energy dissipation capability of the material, increasing the interfacial toughness from approximately 3 J m^−2^ in the native fibrin system to approximately 12 J m^−2^, while simultaneously increasing the burst pressure to 280 mmHg. As a result, the material achieved long-term and stable wound sealing under severe arterial hemorrhage conditions [103]. In addition to introducing exogenous crosslinking networks, regulation of the ionic microenvironment to modulate fibrinogen self-assembly behavior has also gradually emerged as a feasible strategy. Hense et al. challenged the conventional understanding that calcium ions (Ca^2+^) function solely as coagulation cofactors by demonstrating that Ca^2+^ can also serve as an independent regulator of fibrinogen molecular conformation. In the absence of thrombin and factor XIIIa, maintaining Ca^2+^ concentrations within the range of 25–50 mmol/L induced supramolecular assembly of fibrinogen molecules, resulting in the formation of a fibrous pseudo-fibrin hydrogel. This process depended on high-affinity calcium-binding sites within fibrinogen molecules, which promoted fiber formation through electrostatic bridging and conformational regulation. Compared with conventional enzyme-induced fibrin gels, the resulting hydrogel exhibited a denser structure and higher structural stability under alkaline conditions, while simultaneously avoiding the use of costly and unstable thrombin [104]. Furthermore, Anitua et al. compared a sealant derived from autologous platelet-rich plasma (E-sealant) with a commercial high-concentration fibrin glue (Control), revealing the critical influence of fibrinogen concentration on network rheology. These findings suggested that the degradation behavior and mechanical properties of fibrin-based materials can be precisely regulated through adjustment of the molecular microenvironment, thereby providing an important foundation for the development of fibrin sealants with tunable degradation profiles [105].

Overall, protein-based hemostatic materials generally possess well-defined higher-order structures and cell-recognition motifs, such as RGD sequences. Therefore, their molecular modification strategies must carefully balance gelation kinetics, mechanical stability, and preservation of biological activity. In most cases, structural reinforcement is achieved through photocrosslinking or multiple covalent crosslinking strategies. However, excessive crosslinking may generate steric hindrance that masks critical functional motifs, thereby weakening cell–material interactions and impairing tissue repair. Simultaneously, overly dense networks may result in degradation mismatch and induce long-term foreign body responses. In addition, the potential immunogenicity of animal-derived proteins and the complexity of molecular modification processes further increase the difficulty of maintaining batch-to-batch consistency, which remains a major challenge limiting their clinical translation.

## 4. Structural Design of Natural Hemostatic Hydrogels

The macroscopic hemostatic performance of a material is not directly determined by its chemical composition alone, but rather emerges from scaling effects derived from structural organization. From a mechanistic perspective, material morphology originates from the regulation and constraint of intermolecular interactions on assembly pathways. Under the combined influence of interchain interactions, swelling behavior, interfacial energy, blood flow erosion, and physiological environments, materials can develop distinct structural configurations characterized by spatial filling, interfacial anchoring, or network reinforcement. These structural features are subsequently translated into corresponding physical and biological effects within the blood system. Accordingly, the structural construction of natural hemostatic hydrogels can be understood at two interconnected levels. On the one hand, there is the formation of mesoscopic-to-macroscopic structural morphologies, including porous, particulate, and fibrous systems, which mainly regulate blood transport behavior and spatial filling capability. On the other hand, there is the organization of molecular-scale networks and interfacial regulation, such as multicrosslinking and nanocomposite structures, which determine the mechanical stability and environmental responsiveness of the materials. Based on these two dimensions, the structural design strategies of various hemostatic hydrogels can be systematically categorized, as summarized in Table 2, which presents the structural design of hemostatic hydrogels based on natural polymers, thereby enabling further analysis of how structural construction pathways influence hemostatic performance, as well as their applicability and limitations under complex hemorrhagic environments.

### 4.1. Porous Hydrogels

Porous hydrogels achieve hemostatic effects primarily through blood concentration and physical occlusion by constructing highly porous three-dimensional networks, typically with porosities exceeding 90%. Benefiting from their large specific surface area and hydrophilic matrix, these structures generate strong capillary forces upon contact with blood. Once exposed to blood, the micrometer-scale porous network rapidly absorbs plasma water, leading to the local enrichment of erythrocytes, platelets, and fibrinogen within the scaffold. This process lowers the activation threshold of the coagulation cascade and accelerates coagulation progression. Simultaneously, volume expansion induced by fluid absorption generates substantial physical compression, enabling effective filling of irregular wound cavities and sealing of damaged blood vessels. Particularly in deep penetrating wounds and non-compressible hemorrhage (NCH), such three-dimensional porous structures exhibit significantly greater spatial sealing capability than conventional two-dimensional dressings [19]. The formation of porous structures is largely governed by the intrinsic molecular characteristics of the constituent materials, and substantial differences exist among various natural polymers. For example, chitosan and cellulose-derived materials, owing to their abundant hydrogen bonding interactions and relatively rigid molecular chains, readily form stable porous frameworks during freeze-drying processes and are therefore widely used in hemostatic sponges and aerogel systems. In contrast, alginate mainly relies on Ca^2+^-mediated ionic crosslinking, allowing simultaneous gelation and porous structure formation during gas foaming or ion-induced phase separation processes. Protein-based materials such as gelatin, due to their thermoresponsive behavior and reversible gelation properties, are more suitable for generating high-porosity networks through freeze-drying or phase separation methods. These observations indicate that porous structures are not solely determined by fabrication techniques, but are fundamentally constrained by molecular interaction patterns and assembly behaviors at the molecular scale.

#### 4.1.1. Isotropic Porous Structure

**Figure 6:**
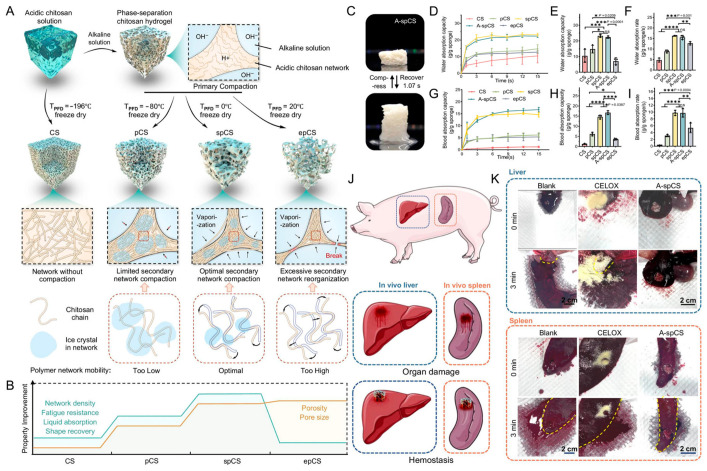
(**A**) Fabrication of sponges by the temperature-assisted secondary network compaction (TA-2ndNC) strategy. The chitosan solution was first treated with phase separation and then subjected to pre-freezing treatments at −80 °C, 0 °C, and 20 °C, followed by freeze drying to fabricate porous chitosan sponge (pCS), superporous chitosan sponge (spCS), and excessively porous chitosan sponge (epCS), respectively. The chitosan sponge (CS) was obtained by freeze-drying the flash-frozen acidic chitosan solution without network compaction. (**B**) The TA-2ndNC strategy was employed to fabricate the sponges with tunable network density, fatigue resistance, liquid absorption capacity, shape recovery ability, pore size, and porosity through the modulation of secondary network reorganization. (**C**) Rapid shape recovery of the alkylated-superporous chitosan sponge (A-spCS) after absorbing water. (**D**–**F**) Water absorption capacity of CS, pCS, spCS, A-spCS and epCS. (**G**–**I**) Blood absorption capacity of CS, pCS, spCS, A-spCS and epCS. (**J**) Schematic diagram of the hemostatic process of A-spCS acting on the pig liver and spleen perforation model. (**K**) Photos of liver/spleen hemostatic effect in blank, CELOX, and A-spCS groups. Statistical significance is indicated as * *p* < 0.05, ** *p* < 0.01, *** *p* < 0.001, **** *p* < 0.0001; ns, not significant. Figures modified from [107] with permission. Copyright 2024, Jiang et al.

The structural design of conventional porous hydrogels primarily focuses on pore size and pore connectivity. Pore size directly determines the sieving and retention behavior of blood components within the porous network. Excessively small pores generate strong physical barriers that restrict blood cell infiltration and subsequently hinder tissue cell migration, whereas excessively large pores weaken capillary forces and reduce blood concentration efficiency. Li et al. investigated cellulose-based hemostatic sponges and demonstrated that coagulation efficiency was strongly dependent on the size of the oriented microchannels. Specifically, sponges with an average pore diameter of approximately 34.2 μm exhibited optimal hemostatic performance, as evidenced by rapid erythrocyte enrichment, accelerated fibrinogen consumption, and enhanced mechanical stability of the formed blood clot. Rheological analysis further indicated that this pore size achieved a favorable balance between fluid absorption rate and local blood retention, thereby facilitating the coagulation process [106]. In addition to pore size, pore connectivity also critically influences blood transport pathways within the material. Jiang et al. developed a temperature-assisted secondary network compaction strategy: primary phase separation is followed by secondary network reorganization at 0 °C, generating superporous chitosan sponges with highly interconnected large pores; modulating this secondary reorganization allows tunable network density, fatigue resistance, liquid absorption capacity, shape recovery, pore size, and porosity (Figure 6A,B). These sponges achieve water-triggered shape recovery in 0.84 s (451% faster than conventional porous sponges), exhibit markedly enhanced water/blood absorption rates and capacity, and retain >95% of maximum stress after 100 cycles (Figure 6C–I). In a Bama minipig non-compressible liver/spleen perforation model, the alkylated version achieves hemostasis in 39 s (338% faster than commercial chitosan powder) and promotes in-situ tissue regeneration (Figure 6J,K) [107]. Furthermore, in situ foaming strategies provide an alternative approach for constructing interconnected macroporous networks. Wang et al. proposed a dual-function pore-forming strategy based on acid-triggered calcium carbonate dissolution, in which the released Ca^2+^ simultaneously promoted alginate ionic crosslinking, while the generated CO_2_ created large-scale porous structures within the hydrogel system. This porous architecture significantly enhanced fluid absorption capacity and improved the transport efficiency of coagulation-related components, while simultaneously promoting platelet aggregation. While maintaining favorable biocompatibility, this system reduced blood loss by up to 77% in complex injury models. Notably, the hydrogel exhibited optimal hemostatic performance when the calcium carbonate-to-alginate ratio reached 40% [108].

#### 4.1.2. Oriented Microchannel Structures

The introduction of anisotropic microchannel architectures provides new structural design concepts for hemostatic sponges. Du et al. constructed a chitosan-based hemostatic sponge containing highly interconnected microchannels by integrating three-dimensional (3D) printed microfiber templating, leaching, freeze-drying, and surface modification techniques. Compared with clinically used gauze, gelatin sponges, and Celox^®^, this sponge exhibited superior procoagulant activity and enhanced hemostatic performance in both normal and heparinized rat and porcine liver perforation models [109].

Inspired by the longitudinal channel structures in Euryale ferox stems, Xie et al. fabricated an anisotropic quaternized cellulose/xanthan gum/reduced graphene oxide (QC/XG/rGO) sponge via directional freeze-casting. The stem-like architecture, featuring highly ordered longitudinal microchannels together with hydrogen bonding, electrostatic interactions, and chemical crosslinking among components, endowed the sponge with excellent elasticity, fatigue resistance, and mechanical compressibility. These aligned channels significantly reduced fluid transport resistance and enabled rapid and efficient liquid absorption. The positively charged and rough surface further promoted aggregation and activation of red blood cells and platelets, as well as fibrin network formation, thereby accelerating blood coagulation. In vivo experiments in rat liver injury, tail amputation, and non-compressible liver hemorrhage models demonstrated superior hemostatic performance compared with commercial gelatin sponge [110]. Building upon this concept, Cao et al. further developed a tapered microchannel structure and demonstrated that such architectures could generate an oriented capillary pressure gradient analogous to that observed in plant xylem systems. As the tapering degree of the microchannels increased, blood transport resistance continuously decreased, thereby significantly shortening hemostatic time [111]. This oriented fluid-guiding architecture fundamentally alters the random liquid transport mechanism characteristic of conventional porous sponges.

#### 4.1.3. Other Hierarchical Structures

Under dynamic physiological conditions and high-pressure hemorrhagic environments, porous materials require more sophisticated spatial architectures to maintain structural stability. Cryogelation and multiscale assembly technologies have therefore emerged as key approaches for constructing highly elastic porous systems. Cryogelation utilizes ice crystals as porogens, concentrating polymer chains within unfrozen microdomains of the liquid phase, where crosslinking subsequently occurs to form dense and mechanically robust pore walls. Such structures endow the material with excellent fatigue resistance and shape-memory properties, allowing the hydrogel to be compressed for injection into narrow wound cavities and to rapidly recover its original morphology upon fluid exposure. For example, the chitosan-based cryogel developed by Qi et al. could be compressed in the dry state and recovered its original shape within approximately 1.7 s after contact with liquid, while exhibiting a fluid absorption ratio of nearly 41-fold [112]. To further reinforce pore-wall mechanical strength, Li et al. incorporated critical-size calcium phosphate (C–CaP) nanostructures with high flexural strength into a sericin sponge, thereby constructing an organic–inorganic composite porous architecture. This structural design enabled the material to simultaneously exhibit a high elastic modulus of 36.0 kPa, a rapid fluid absorption rate of 1.2 g·cm^−3^·s^−1^, and a high spatial filling efficiency of 200.0%·s^−1^ [113].

At the macroscopic level, the introduction of multiscale and gradient structures provides new strategies for improving material adaptability under complex loading environments. Cai et al. utilized bidirectional freezing to fabricate a cellulose sponge with an arched layered structure, in which calcium ions formed stable interlayer supports, enabling the material to maintain structural integrity under compression while achieving approximately 11-fold volume expansion following fluid absorption. Consequently, the sponge could provide sustained and uniform compressive force during hemostasis [114]. Inspired by the nanofibrous architecture of natural blood clots, Zhang et al. further designed a QCS nanofiber sponge with a gradient pore-size distribution. In this system, the small pores in the outer layer efficiently intercept blood cells, achieving a filtration efficiency of 91.7%, whereas the larger pores in the inner layer enabled rapid plasma absorption with a capacity of 93 g/g. In a rat liver defect model, the hemostatic time of this material was approximately 2.5-fold shorter than that of commercially available Gelatamp^®^ hemostatic sponges [115]. Furthermore, the direct utilization of decellularized extracellular matrix (dECM) enables the construction of porous systems possessing multilevel biomimetic characteristics. These materials preserve the complex compositions of proteins, polysaccharides, and proteoglycans present in native tissues, thereby facilitating tissue repair and regeneration. The kidney-derived decellularized extracellular matrix (kdECM) porous sponge developed by Kim et al. significantly improved renal wound regeneration while simultaneously achieving rapid hemostasis [116]. Most current studies focus on optimizing local properties through adjustment of individual parameters, such as pore size or crosslinking density. However, such approaches frequently compromise structural stability or tissue adaptability. From the perspective of structural construction, porous hydrogels inherently face a trade-off among fluid absorption efficiency, mechanical stability, and tissue compatibility. Achieving a balance among these factors, therefore, represents the primary challenge in the optimization of porous hemostatic hydrogels. More importantly, this limitation also indicates that current structural design strategies still largely rely on empirical optimization and lack predictable structure–function relationship models. Consequently, future development should emphasize multiscale structural co-design to establish quantitative correlations between structural parameters and hemostatic performance, thereby promoting the transition of material design toward a mechanism-driven paradigm.

### 4.2. Particle-Based Hydrogels

In this review, discrete material forms such as microspheres, particles, and powders are collectively categorized as particle-based systems. In these systems, independent particulate units participate in transport, aggregation, and structural rearrangement at the bleeding site to achieve hemostatic functionality. Unlike porous hydrogels, which rely on preconstructed continuous networks, particle-based systems mainly depend on the spontaneous reorganization capability of discrete units within dynamic physiological environments. This structural feature endows such materials with excellent injectability and spatial adaptability, enabling their delivery through catheters into deep wound cavities or narrow hemorrhagic sites, where adaptive filling of irregular spaces can subsequently be achieved [117,118]. However, because the structural formation of these systems is strongly triggered by external environmental conditions, their practical applications are often associated with pronounced environmental dependence and dynamic uncertainty. Furthermore, the formation of particle-assembled hydrogels largely depends on the dispersion stability of particles in solution and their interfacial interaction characteristics. The assembly process is jointly regulated by molecular chain dissolution behavior, crosslinking kinetics, and phase separation properties. Specifically, alginate, owing to its rapid Ca^2+^-mediated ionic crosslinking capability, can readily form stable microspheres under emulsion or spray-processing conditions and therefore represents a typical matrix for particle-based assembly systems. Chitosan and its derivatives, benefiting from tunable charge density and hydrogen-bonding interactions, can easily generate functional particles with strong interfacial adhesion capability within microfluidic or reverse-emulsion systems. HA and certain modified starch systems, due to their high hydrophilicity and dynamic covalent bonding characteristics, are more suitable for constructing injectable systems capable of transitioning from discrete particles into continuous network structures. In contrast, protein-based materials such as fibrin and collagen exhibit a stronger tendency to form continuous networks or fibrous structures and therefore possess relatively limited applicability in particle-based systems.

#### 4.2.1. Self-Gelling Powders

**Figure 7:**
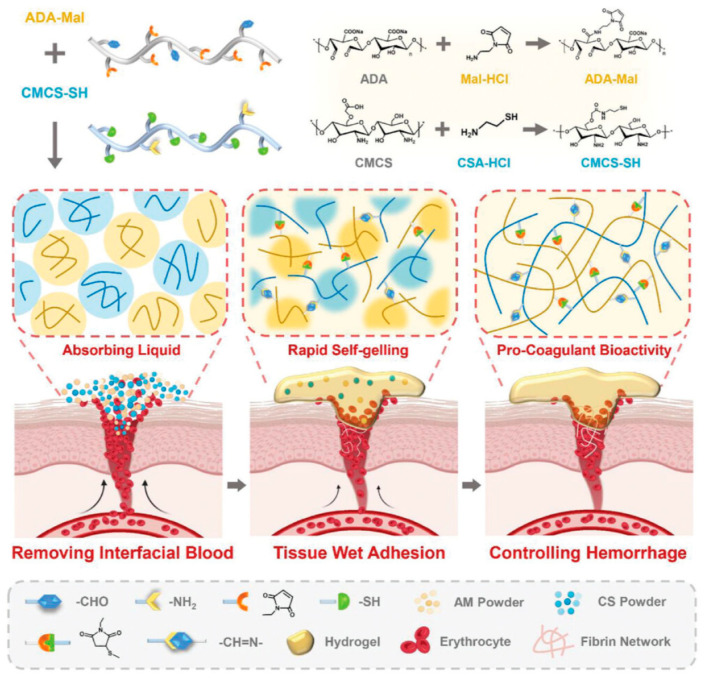
Schematic representation of the chemical synthesis process of SA grafted aldehyde and maleimide groups (ADA-Mal) and CMCS grafted sulfhydryl group (CMCS-SH), and the preparation of rapid self-gelling powder for the control of non-compressible massive hemorrhage. Asterisks (*) denote the continuation of the polymer backbone. Reprinted with permission from ref. [119]. Copyright 2023 Lei et al. Advanced Science is published by Wiley-VCH GmbH.

To address the tendency of discrete particles to disperse under high blood-flow conditions, recent research has increasingly focused on self-gelling powder systems. These materials exist as free-flowing powders in the dry state but can rapidly undergo physical or chemical crosslinking upon contact with blood, thereby forming continuous adhesive hydrogel networks in situ. Lei et al. developed a biopolymer-based powder for the control of non-compressible hemorrhage (Figure 7). Upon contact with blood, it rapidly absorbs fluid, dissolves, and exposes active functional groups, followed by sequential cross-linking via thiol–Michael addition and Schiff base reactions, enabling self-gelation within 10 s and effective wound sealing to achieve hemostasis. The rapid removal of interfacial tissue fluid and strong material–tissue interactions ensure reliable wet adhesion and sealing, resulting in significant hemostatic efficacy in non-compressible severe hemorrhage models in rats, rabbits, and beagle dogs [119]. The sodium aescinate self-gelling powder (SA self-gel-P) developed by Li et al. reorganized into a continuous network through intermolecular hydrogen-bond-mediated β-sheet structures upon blood exposure, thereby simultaneously achieving synergistic drug release and hemostatic functionality [120]. In another study, Zhang et al. utilized the multiple intermolecular interactions among TA, chitosan, and polyethylene glycol (PEG) to induce aggregate formation through liquid–liquid phase separation (LLPS), thereby enabling a structural transition from discrete particles into an integrated bulk hydrogel network [30].

#### 4.2.2. Microspheres with Advanced Structures

The hemostatic mechanism of microsphere-based systems can be understood as a swelling-induced jamming process, in which particles rapidly transition from a dispersed fluid-like state into a densely packed structure. During this process, volume expansion generates compressive pressure that facilitates vascular sealing. Building upon this mechanism, modern microsphere designs increasingly integrate hierarchical porous architectures and biomimetic structures to enhance zeolite-like adsorption effects and increase specific surface area, thereby improving the enrichment efficiency of coagulation-related components [121,122]. Beyond physical blood absorption and concentration, SF-based microdroplet systems have also demonstrated considerable potential as carriers for bioactive factor stabilization and controlled delivery. Using a microfluidic flow-focusing strategy, monodispersed SF microdroplets were successfully fabricated for horseradish peroxidase (HRP) encapsulation, significantly improving protein stability during long-term storage. The β-sheet-associated microenvironment of SF not only protected the bioactivity of encapsulated proteins but also provided structural uniformity advantageous for controllable release behavior. These findings suggest that SF-based particulate systems may serve as multifunctional platforms integrating hemostasis with localized therapeutic factor delivery [123]. Xi et al. reported a polysaccharide hemostatic microsphere (PHM) possessing a lotus pod-like surface morphology. The surface pits guided blood rapidly into interconnected internal channels, while the gradually narrowing pores continuously concentrated coagulation-related components within the microspheres during fluid transport. The material achieved a fluid absorption rate of 40.7 mL·s^−1^·cm^−2^, significantly reducing the in vivo hemostatic time from 210 s to 45 s [124]. Ouyang et al. utilized rod-like cellulose nanocrystals as structural scaffolds and fabricated sodium alginate/cellulose nanocrystal porous microspheres (SA/CNC) through a reverse-emulsion method. The resulting microspheres exhibited high porosity, excellent fluid absorption capability, and favorable hemostatic performance [125]. Similarly, hierarchically structured hollow starch microparticles (Ca@MSMP) and chitosan/β-glycerophosphate (CS/β-GP) microspheres prepared through microencapsulation strategies both enhanced fluid absorption and blood cell adsorption through specifically designed microscale porous structures [126,127]. Compared with conventional macroporous sponges, microsphere systems combine highly efficient blood concentration capability with excellent injectable flow properties.

#### 4.2.3. Functionally Integrated Microspheres

Particle-based systems are susceptible to reflux and distal dispersion during blood flow, which may compromise local hemostatic efficacy. To overcome this limitation, various functional integration strategies have been explored. Li et al. developed thrombin-modified poly(vinyl alcohol)/chitosan (PVA/CS) composite microspheres. Under blood-flow conditions, surface-immobilized thrombin accelerated the local coagulation process, thereby improving particle retention at the bleeding site. In addition, incorporation of barium sulfate (BaSO_4_) nanoparticles into the microsphere core endowed the material with X-ray visibility, enabling precise delivery and real-time monitoring during interventional procedures. This multifunctional design integrating imaging and coagulation regulation significantly improved both the controllability and safety of the hemostatic process [128]. Self-propelled particle systems have also gradually attracted attention for applications involving deep blind-cavity injuries or retrograde high-pressure blood flow, where passive transport is insufficient. Zhao et al. designed an injectable hydrogel adhesive capable of autonomous expansion and self-propulsion. By utilizing the reaction between porous calcium carbonate (CaCO_3_) and acetic acid to generate CO_2_ within the system, the thrust generated by gas expansion could overcome blood flow resistance, enabling the material to actively penetrate into the blind end of the wound cavity and achieve autonomous filling [129]. This strategy fundamentally shifts particle-based systems from passive accumulation toward active structural construction capability. Future development of particle-based hemostatic systems should focus on achieving controllable degradation behavior and on-demand removability. Overall, particle-based hemostatic hydrogels require synergistic optimization of transport behavior, structural reorganization, and degradation kinetics in order to balance rapid hemostatic efficacy with long-term biosafety.

### 4.3. Fibrous Hydrogels

The ultimate product of physiological hemostasis in the human body is a fibrin clot formed through the enzymatic assembly of fibrinogen into a nanoscale fibrin network [135]. Owing to their high specific surface area and distinct nanotopological characteristics, fibrous structures can effectively capture blood cells while promoting platelet activation and platelet aggregation. This phenomenon indicates that fibrous morphologies play a critical role in regulating cellular behavior and the coagulation process [130,131]. Natural polymers can form fibrous units ranging from the nanoscale to the microscale through intermolecular interactions, such as intrinsic secondary structures (e.g., β-sheet structures), or through external fabrication strategies, thereby further constructing multiscale fibrous networks [20,132]. Compared with porous structures and particle-based systems, the formation of fibrous hydrogels relies more strongly on the ability of molecular chains to undergo ordered alignment along a one-dimensional axis. Consequently, fibrous structural assembly imposes stricter requirements on molecular conformation and intermolecular interaction mechanisms. The formation of fibrous architectures is intrinsically constrained by molecular conformation and assembly pathways. Silk fibroin, owing to its repetitive Gly–Ala sequences, can spontaneously self-assemble into stable β-sheet structures and naturally possesses the capability to form nano- and microscale fibers, making it a representative material for fibrous network construction. In contrast, collagen and gelatin depend on triple-helix structures or partially retained structural motifs to form bioactive fibrous networks through electrospinning or induced self-assembly processes. Fibrin, as the terminal product of physiological hemostasis, inherently exhibits a nanofibrous architecture and can directly form fibrous networks in vivo through enzymatic reactions.

#### 4.3.1. In Situ Fibrous Networks

In situ fibrous networks refer to nanofibrous structures that are directly formed through molecular self-assembly or biological synergistic assembly within body fluids or blood environments. A defining feature of these systems is that structural formation occurs during application rather than through prefabrication. Among them, supramolecular self-assembly utilizes hydrogen bonding, π–π interactions, and electrostatic interactions to drive molecular alignment and nanofiber formation within physiological environments, representing an important strategy for constructing biomimetic ECM-like structures. Peptides, as the fundamental functional units of proteins, are representative materials for such systems. The typical ion-complementary short peptide RADA16 can rapidly self-assemble within less than 15 s upon contact with blood ions, forming a nanofibrous network with a diameter of approximately 10 nm [133]. Such materials possess high water content and excellent optical transparency, making them particularly suitable for minimally invasive surgical procedures requiring real-time visual observation [134,135]. Building upon this strategy, the degradation behavior and environmental responsiveness of these materials can be further regulated through sequence engineering. Chaugule et al. constructed three types of microcollagen possessing highly similar amino acid sequences but differing numbers of matrix metalloproteinase-1 (MMP-1) cleavage sites. Their results demonstrated that adjusting the number of cleavage sites or modifying key amino acid residues enabled approximately two orders of magnitude variation in degradation rates under MMP-1-mediated enzymatic degradation, thereby providing a novel strategy for temporally controlled functional regulation of biomimetic ECM scaffolds [136]. Polysaccharide-based materials, such as alginate and HA, generally tend to form amorphous gels or particulate structures because they lack stable secondary structures and oriented assembly capability. Nevertheless, several studies have demonstrated that polysaccharides can also be induced to form fibrous architectures through supramolecular assembly strategies. Wang et al. reported that an alginate-based system formed nanofibrillar hydrogels through hierarchical self-assembly. This fibrillar architecture endowed the hydrogel with excellent mechanical properties, shear-thinning recovery behavior, and outstanding in vivo hemostatic performance [137], indicating that natural polysaccharide systems also possess the potential to construct in situ fibrous networks.

Furthermore, some materials can undergo synergistic assembly with the endogenous coagulation system. The amphiphilic peptide designed by Padilla–Lopategui et al. could specifically crosslink with coagulation factor XIIIa and co-assemble with the endogenous fibrin network during coagulation, thereby forming an enhanced nanofibrous composite hydrogel. This fibrous structure, synergistically constructed from both peptide molecules and blood components, not only mimicked the microenvironment of natural hematomas but also actively regulated physiological hemostasis and tissue repair processes [138]. A key characteristic of such systems is that the material directly participates in the endogenous coagulation process, thereby achieving synergistic structural construction with the native blood system.

#### 4.3.2. Preformed Fibrous Networks

Preformed fibrous systems refer to materials in which fibrous architectures are fabricated in vitro prior to application and subsequently participate in the hemostatic process in a predefined structural configuration. Unlike in situ fibrous systems, the structures of these materials are fixed before use, and their hemostatic function does not rely on further self-assembly to generate continuous networks during application. Among natural protein-based systems, the polycationic filaggrin materials reported by Wang et al. were designed to mimic the aggregation behavior and conformational transitions of fibrin. These materials not only exhibited excellent shape-memory recovery and mechanical reinforcement properties, but also promoted hemostasis and tissue regeneration through regulation of platelet-associated signaling pathways, demonstrating superior performance compared with clinically used materials in complex hemorrhagic models [139]. Polyelectrolyte complex (PEC) systems also belong to this category. Mishra et al. utilized electrostatic interactions between chitosan and casein to construct nanofibrous composite structures in vitro. The fiber diameter, surface characteristics, and fluid absorption capacity of these materials could be regulated through adjustment of fabrication parameters [140]. In vivo studies demonstrated that the material achieved rapid hemostasis within approximately 9 s; however, because its mechanism does not involve dynamic inter-fiber network reorganization, it is more appropriately classified as a preformed fibrous unit participating directly in hemostasis. Electrospun nanofibrous materials also represent typical examples of preformed fibrous systems. Parhi et al. introduced an ultrathin polycaprolactone/keratin (PCL/keratin) nanofibrous membrane onto the surface of conventional gauze. Benefiting from its high specific surface area, this structure effectively promoted platelet adhesion, platelet aggregation, and platelet activation. Simultaneously, the nanofibrous architecture induced polyhedral deformation of erythrocytes, enabling tighter cellular packing and formation of a denser physical barrier, thereby reducing blood leakage [141]. In such systems, fibrous structures are directly generated through external fabrication techniques, and their enhanced functionality mainly originates from optimization of surface topology and interfacial characteristics. At a more advanced level, preformed fibrous architectures can also be engineered through spatial structural design and multilayer electrospinning strategies to generate asymmetric Janus structures with multifunctional integration capability. Mehmood et al. fabricated a bi-layered Janus fibrous hydrogel dressing consisting of a zinc-doped silica-reinforced catechol gelatin bottom layer for wet-tissue adhesion, hemostasis and antibacterial activity, and a zwitterion-functionalized cellulose acetate top layer for antifouling and postoperative anti-adhesion [142]. This hierarchical architecture enabled unidirectional transport of wound exudates, thereby improving the local wound fluid environment while maintaining effective hemostatic performance, ultimately helping to reduce infection risk and promote wound healing. Beyond direct hemostatic regulation, fibrous SF-based architectures also exhibit considerable potential for vascularized tissue reconstruction. A three-dimensional SF microsphere–nanofiber scaffold fabricated through the combination of electrospinning and microfluidics was shown to mimic the native extracellular matrix microenvironment more effectively than conventional porous sponges. The bridge-like hierarchical architecture promoted endothelial cell proliferation and preserved the expression of vascular-related markers, including CD146, VE-cadherin, and PECAM-1. Such findings indicate that microsphere–nanofiber hybrid structures may simultaneously reduce thrombosis risk and facilitate post-hemostatic vascular remodeling [143].

Although fibrous structures possess unique advantages in biomimetic interface construction and cellular regulation, they still exhibit several limitations in rapid hemostatic applications. For systems relying on in situ self-assembly, if the fibrous network formation rate is slower than blood flow erosion under high-flow hemorrhagic conditions, continuous barriers cannot be established rapidly enough to achieve effective hemostasis. Conversely, although preformed fibrous materials possess high specific surface areas, they generally lack substantial volume expansion and spatial filling capability, making it difficult to achieve effective sealing in large-volume or deep wound cavities. Furthermore, under continuous external mechanical loading, fibrous networks may undergo fiber slippage or structural rearrangement, thereby compromising overall structural stability.

### 4.4. Multicrosslinked and Multinetwork Hydrogels

A material’s resistance to blood flow erosion primarily depends on the mechanical properties of its internal polymer network. Traditional single-network hydrogels generally suffer from structural fragility under high-water-content conditions. Following fluid absorption and swelling, their mechanical strength decreases markedly, rendering them susceptible to rupture or interfacial delamination and therefore incapable of withstanding physiological arterial pressure. To overcome these limitations, current research mainly follows two strategies. One strategy involves introducing multiple crosslinking mechanisms within a single network to construct synergistic energy-dissipation systems through multicrosslinking. The other strategy focuses on increasing the number of networks to form multinetwork architectures, including interpenetrating or hierarchical network structures, thereby comprehensively enhancing both mechanical properties and functional performance [144,145,146]. Furthermore, the integration of multinetwork and multicrosslinking strategies enables hydrogels to simultaneously achieve mechanical stability and interfacial adaptability under complex hemorrhagic conditions.

#### 4.4.1. Physicochemical Dual-Crosslinking Networks

Introducing different types of crosslinking interactions into a single-polymer system represents a fundamental strategy for improving the mechanical properties of hydrogels. Reversible physical crosslinks, including hydrogen bonding, ionic coordination, or crystalline domains, preferentially dissociate under external stress to dissipate energy, whereas stable chemical crosslinks provide long-term structural support. Together, these interactions establish a synergistic energy-dissipation network. To address the brittle fracture behavior and poor recovery capability of conventional porous sponges following compression, Lin et al. developed a fully cellulose-based super-expandable dual-crosslinked sponge. In this system, chemical crosslinking was achieved through covalent acetal bonds formed by glutaraldehyde, while a freeze-induced hydrogen-bonding network was simultaneously introduced to construct a stable three-dimensional porous architecture. Upon contact with blood, the material expanded to approximately 3900% of its original height and demonstrated excellent hemostatic performance in a porcine femoral artery hemorrhage model [147]. Similarly, Jiang et al. constructed a hierarchical aerogel by integrating a covalently crosslinked framework with a freeze-induced hydrogen-bond network. This material maintained structural integrity even under an extreme compressive strain of 90% and exhibited excellent fatigue resistance and shape-memory capability over 1000 compression cycles. In practical applications, its elastomer-like mechanical response enabled adaptation to dynamic deformation in regions such as joints, while its fluid absorption rate was significantly higher than that of commercially available polyurethane (PU) foam. Moreover, the material did not induce significant immune responses throughout the degradation process [148]. Overall, physicochemical dual-crosslinking strategies integrate reversible and stable intermolecular interactions to synergistically enhance both energy dissipation and structural stability under wet conditions, thereby effectively addressing the brittleness limitations associated with conventional hydrogels.

#### 4.4.2. Multinetwork Structures

Multinetwork hydrogels are generally formed through the interpenetration of networks possessing distinct compositions or kinetic characteristics. In a typical double-network design, the system relies on the synergistic interaction between a rigid sacrificial network and a flexible supporting network, thereby effectively dissipating mechanical energy under external loading while preserving overall structural integrity. The construction of interpenetrating networks using natural polymers can avoid the potential biosafety concerns associated with synthetic monomers. Liu et al. designed an injectable double-network hydrogel based on dialdehyde-modified carboxymethyl cellulose (DCMC) and gelatin. This system simultaneously incorporated Schiff base crosslinking and amide crosslinking structures, in which the amide network provided fundamental mechanical support, while the Schiff base network endowed the material with self-healing capability and wet adhesion properties. The hydrogel underwent in situ gelation within approximately 8 s and demonstrated superior hemostatic performance compared with single-network systems in liver injury and tail-amputation hemorrhage models [145]. Furthermore, the introduction of a third network can further enhance mechanical performance while expanding functional diversity. Chen et al. incorporated poly(γ-glutamic acid) (γ-PGA) into a dual-network system composed of CMCS and oxidized dextran (ODex), thereby constructing a triple-network hydrogel architecture. Owing to its strong hydrophilicity, γ-PGA rapidly removed moisture from the wound interface, thereby strengthening interfacial adhesion between the hydrogel and tissue. The resulting triple-network hydrogel achieved a burst pressure of 238 mmHg, exceeding the systolic blood pressure of healthy adults, and effectively sealed large-area liver injuries [149]. In addition, multinetwork systems integrating natural polysaccharides and protein materials can be combined with advanced fabrication technologies to construct complex architectures while maintaining excellent biocompatibility. For example, a recently reported double-network hydrogel suitable for three-dimensional (3D) printing was composed of oxidized hyaluronic acid, acylhydrazide-modified hyaluronic acid, and cold-water fish gelatin. This system combined the fluid absorption capability of hyaluronic acid with the cellular affinity of gelatin, while gallic acid modification further improved its mechanical properties, thereby overcoming the insufficient mechanical strength typically observed in conventional hydrogels [150]. Overall, multinetwork structural design achieves a balance between mechanical reinforcement and multifunctional synergy through increasing network hierarchy and integrating complementary functions.

#### 4.4.3. Dynamic Reconfigurable Networks

In dynamic physiological environments such as the heart or large blood vessels, hemostatic materials must not only withstand high pressure but also accommodate continuous cyclic deformation. Consequently, the incorporation of dynamic reversible interactions to construct reconfigurable networks—thereby endowing materials with breakable-recoverable structural behavior under mechanical loading—has become an important design strategy. Zhang et al. developed an injectable dual-network adhesive hydrogel (DNAH) integrating radical polymerization with Schiff base chemistry, thereby constructing a system that simultaneously possessed high mechanical strength and time-dependent adhesive behavior. This material utilized the redox responsiveness of catechol groups to dynamically regulate functionality. During the initial hemostatic stage, catechol groups provided strong wet adhesion to tissue, enabling the hydrogel to achieve a burst pressure exceeding 280 mmHg. Over time, however, catechol groups gradually oxidized into quinone structures, resulting in a reduction in surface adhesion. Consequently, this system enabled rapid sealing of penetrating cardiac injuries during the acute phase while reducing the risk of postoperative tissue adhesions during later stages, thereby minimizing complications associated with excessively adhesive traditional materials [151]. To address fatigue fracture under dynamic loading conditions, Yan et al. proposed a dual-dynamic-bond synergistic strategy for constructing a highly adhesive hydrogel system. Its network architecture was formed through the synergistic interaction of two physical crosslinking mechanisms. The first network consisted of abundant reversible hydrogen bonds formed between the polyphenolic hydroxyl groups of TA and the carboxyl groups of poly(acrylic acid), whereas the second network was composed of ionic coordination structures formed between sodium alginate and calcium ions. Under cyclic loading, the continuous dissociation and reformation of these reversible interactions enabled effective energy dissipation while maintaining structural integrity. Experimental results demonstrated that this hydrogel effectively achieved hemostasis in rat cardiac and femoral artery hemorrhage models, exhibiting excellent mechanical stability and hemostatic performance under dynamic high-pressure physiological conditions [152]. Overall, the incorporation of dynamic reversible interactions has driven the evolution of multinetwork hydrogels from simple mechanically reinforced materials into adaptive structural systems possessing environmental responsiveness. More broadly, the design of multicrosslinked and multinetwork hydrogels has progressively shifted from basic mechanical enhancement toward comprehensive regulation of network topology, crosslinking kinetics, and energy-dissipation behavior. Through the integration of physicochemical crosslinking, multinetwork architectures, and dynamic reconfigurable mechanisms, these materials can effectively balance mechanical strength, structural stability, and interfacial adaptability under complex hemorrhagic conditions, thereby providing reliable mechanical support for wound hemostasis.

### 4.5. Nanocomposite Hydrogels

The incorporation of nanoscale components into hydrogel systems has emerged as an important strategy for enhancing the comprehensive performance of natural hemostatic hydrogels [153]. These nanophases can function either as reinforcing components to improve the overall mechanical properties of the matrix or as active participants in regulating blood components during hemostasis. This section primarily focuses on the roles of nanofillers in matrix mechanical reinforcement and physical activation at the hemostatic interface.

#### 4.5.1. Mineral-Based Nanophases

To address the common limitations of natural polysaccharide hydrogels, including insufficient mechanical strength and limited wet adhesion capability, the introduction of silicate-based nanomaterials has become a widely adopted modification strategy. These mineral nanocomponents possess extremely high specific surface areas and unique layered charge distributions, enabling them to enhance the network modulus through skeletal reinforcement while simultaneously serving as physical crosslinking sites that restrict molecular chain slippage. Among two-dimensional layered minerals, Huang et al. developed a chitosan/organic rectorite (OREC) composite hydrogel (GCCO) and further incorporated a gelatin/β-cyclodextrin matrix. The rectorite nanosheets functioned as rigid structural nodes that immobilized chitosan molecular chains through electrostatic interactions, thereby increasing the tensile strength of the hydrogel to 41.3 ± 3.56 kPa while maintaining porous structural integrity under humid conditions [154]. Beyond structural reinforcement, nanoscale Laponite also contributes to activation of the endogenous coagulation cascade through its negatively charged surface. Studies have demonstrated that synergistic incorporation of Laponite with polydopamine (PDA) into modified cellulose sponges enabled a wet adhesion strength of 405 kPa and produced superior hemostatic performance compared with control materials in multiple high-risk hemorrhage models [155]. With respect to one-dimensional nanostructures, rod-like minerals such as attapulgite can form microfiber-like reinforcing architectures within polymer networks. Through hydrogen bonding and electrostatic interactions, these nanostructures establish dense reinforcing networks, thereby significantly improving the mechanical strength and toughness of the hydrogel system [156].

#### 4.5.2. Carbon-Based Nanophases

Compared with rigid inorganic nanomaterials, carbon-based nanomaterials exhibit unique advantages in regulating flexibility and interfacial properties. Carbon dots (CDs) are zero-dimensional nanomaterials typically smaller than 10 nm in diameter, with surfaces enriched in hydrophilic functional groups capable of forming multiple intermolecular interactions with polymer chains, thereby optimizing network architecture. In one study, okra-derived carbon dots were incorporated into a chitosan/poly(vinyl alcohol) (CS/PVA) sponge system. Scanning electron microscopy (SEM) analysis demonstrated that the carbon dots promoted the formation of an interconnected porous architecture through hydrogen-bond-mediated interactions. This structure endowed the sponge with excellent flexibility and a fluid absorption capacity reaching several tens of times its own weight, enabling close adaptation to irregular wound surfaces while effectively promoting hemostasis and wound healing [157]. Among two-dimensional carbon-based nanostructures, graphene oxide (GO) and its reduced derivative, reduced graphene oxide (rGO), represent key components for enhancing both mechanical properties and interfacial functionality. Owing to their exceptionally large two-dimensional specific surface areas, GO/rGO nanosheets can establish dense crosslinked interactions with polymer chains, thereby significantly enhancing the burst-pressure resistance of hydrogels. A recent study reported a mussel-inspired polydopamine-grafted reduced graphene oxide/alginate (PDA-rGO/Alg) composite hydrogel. In this system, alginate (Alg) functioned as the hydrophilic network scaffold, whereas the two-dimensional PDA-rGO nanosheets reinforced the hydrogel matrix, resulting in a 3.5-fold increase in storage modulus (G’). Simultaneously, the material established continuous conductive pathways and achieved a conductivity of 3.95 mS·cm^−1^, closely matching the electrophysiological microenvironment of human skin. This feature not only contributed to stable performance under dynamic physiological conditions but also provided potential applications in wound monitoring and electrostimulation-assisted therapy [158].

#### 4.5.3. Other Nanophases

For more complex hemorrhagic scenarios, such as bone defects or deep wounds accompanied by high-pressure bleeding, the incorporation of bioceramic or metal–organic hybrid nanocomponents can endow hydrogels with multiple bioactive functionalities. Beyond serving as structural reinforcing phases, these nanoscale units can also participate in network crosslinking, drug delivery, and biological regulation, thereby enabling synergistic integration of structural and functional properties. In hard-tissue hemorrhagic environments, the incorporation of HAP nanoparticles significantly enhances the mechanical support capability of hydrogels while simultaneously providing osteogenic and antibacterial activity, thereby satisfying the dual requirements of hemostasis and tissue repair [159]. Zhao, Yuan, and colleagues introduced metal-coordinated polymer nanoparticles (ICPs) composed of proanthocyanidins, berberine hydrochloride, and Fe(III) into a hydrogel system based on oxidized chondroitin sulfate (OCS) and carboxymethyl chitosan (CMC). These nanoparticles functioned not only as drug delivery carriers but also as dynamic coordination crosslinking sites within the network, thereby endowing the hydrogel with enhanced self-healing capability and improved mechanical strength. Meanwhile, their antibacterial activity, anti-inflammatory regulation, and tissue-regenerative properties contributed to improved therapeutic outcomes in complex wound environments [160].

Although nanocomposite strategies exhibit significant advantages in enhancing the mechanical performance and multifunctional integration of hydrogels, their application in extreme hemorrhagic conditions still faces substantial hematological and pathological challenges. One major concern is hemocompatibility and the associated risk of systemic embolization. Under conditions of arterial hemorrhage or massive visceral bleeding, inorganic or metallic nanoparticles that are not effectively retained within the hydrogel network may enter systemic circulation through the bloodstream, potentially causing endothelial injury, inducing disseminated intravascular coagulation (DIC), or forming ectopic microemboli within vital organs. In addition, high-velocity blood flow imposes greater demands on the local retention capability of nanocomponents. Once nanoparticle migration or leakage occurs, local procoagulant efficiency may decrease substantially while systemic toxic side effects may become amplified. Furthermore, acute hemorrhagic microenvironments are often accompanied by pH fluctuations, competitive protein adsorption, and accumulation of ROS, all of which may induce nanoparticle aggregation or alter surface physicochemical properties, thereby impairing wet adhesion and procoagulant activity at the interface. Therefore, the design of nanocomposite hemostatic hydrogels must carefully balance coagulation-promoting capability with controllable in vivo clearance behavior. Moreover, systematic evaluation frameworks integrating blood rheology, nanomaterial toxicology, and in vivo metabolic behavior are required to support the safe clinical translation of such materials. Overall, this section systematically summarizes multiscale structural design strategies for natural polymer-based hemostatic hydrogels. Through hierarchical spatial organization and structural upscaling, different structural design approaches transform molecular-scale interfacial interactions into macroscopic functions capable of operating under complex blood-flow conditions. Nevertheless, the effectiveness of this transformation process remains fundamentally constrained by both the persistence of interfacial interactions and the long-term stability of the constructed structures.

## 5. Advanced Functionalities of Hydrogels

Through the aforementioned molecular engineering and structural design strategies, natural polymer-based hemostatic hydrogels can effectively achieve rapid bleeding control and stable wound sealing under complex hemorrhagic conditions. Beyond acute hemostasis, recent research has increasingly focused on integrating therapeutic and bioactive functions into these materials to support subsequent wound healing and tissue regeneration processes. In clinical settings, particularly in diabetic wounds, infected injuries, and deep visceral trauma, the local microenvironment often exhibits persistent inflammation, oxidative stress, bacterial infection, and impaired tissue regeneration following initial hemostatic stabilization. Under such conditions, effective hemostasis alone is insufficient to ensure favorable clinical outcomes. Instead, long-term therapeutic efficacy increasingly depends on the material’s ability to regulate coagulation activity, prevent infection, modulate the immune microenvironment, and promote tissue repair. Meanwhile, intelligent sensing and adaptive therapeutic intervention based on pathological microenvironmental changes are emerging as important directions in precision biomaterials. Accordingly, this section systematically summarizes recent advances in bioactive and adaptive therapeutic strategies for natural polymer-based hemostatic hydrogels, including coagulation regulation, antibacterial activity, immune microenvironment modulation, tissue regeneration, as well as dynamic monitoring and stimulus-responsive intervention, as summarized in Table 3, which presents advanced functionalities of natural polymer-based hemostatic hydrogels.

### 5.1. Coagulation Modulation and Hemostatic Enhancement

Under pathological conditions such as coagulopathy, impaired platelet activity, or extreme hemorrhage, actively regulating the coagulation process at the biochemical level through functionalized material design represents an effective strategy for improving clinical applicability. Compared with passive hemostatic mechanisms that primarily depend on the intrinsic physicochemical properties of the material, functionalized hydrogels can serve as delivery platforms for bioactive agents, thereby intervening in and modulating different stages of the coagulation cascade through the incorporation of exogenous procoagulant components. Current research in this field mainly focuses on three aspects: promoting initiation of the coagulation cascade, accelerating coagulation reaction kinetics, and enhancing clot stability while inhibiting the fibrinolytic system.

#### 5.1.1. Enhancement of Coagulation Cascade Initiation

During the early stage of acute hemorrhage, rapid activation of the intrinsic coagulation pathway is a prerequisite for effective hemostasis. Inorganic mineral-based materials, owing to their high specific surface area and negatively charged surfaces, can specifically adsorb and activate coagulation factor XII (FXII, Hageman factor), thereby triggering the intrinsic coagulation cascade. The kaolin–zeolite composite hemostatic system developed by Zhang et al. exhibits a characteristic cascade-amplification effect. In this system, kaolin primarily mediates FXII activation, whereas zeolite further concentrates activated coagulation factors, including FXa and FVa, through its porous structure, thereby promoting the generation of highly active thrombin. The functional division and synergistic cooperation between these two components substantially enhance the overall efficiency of the coagulation cascade, enabling the material to reduce blood loss by 75% and shorten hemostasis time by 33% in a rabbit femoral artery hemorrhage model. Moreover, this kaolin–zeolite gauze exhibits negligible heat-generation effects and an almost zero particle-shedding rate, demonstrating improved biosafety compared with currently available commercial inorganic hemostatic gauzes [161]. Building on this strategy, incorporation of inorganic components into polymeric networks to construct composite hydrogels has proven effective for enhancing hemostatic activity while preserving structural integrity [162,163]. For example, Song et al. incorporated kaolin into a carboxymethyl chitosan/sodium alginate composite sponge system and established a sustained calcium-ion release platform using glucono-δ-lactone (GDL) and Ca-EDTA, thereby achieving synergistic enhancement of structural support and coagulation activation. This material demonstrated superior hemostatic performance compared with commercially available Gelatamp^®^ gelatin sponges in mouse models of tail amputation, femoral vein injury, and liver injury [164]. In addition, mesoporous bioactive glass (MBG), owing to its nanoscale surface architecture and ion-release capability, can promote rapid protein corona formation and amplify thrombin generation, thereby accelerating fibrin network formation. Wu et al. immobilized nanoscale MBG onto cotton fabric surfaces and further pre-coated the particles with ε-polylysine and alginate to develop an advanced dressing. The immobilized MBG layer markedly enhanced the adhesion, aggregation, and activation of red blood cells and platelets, while the rapid formation of a dense fibrin network on the MBG surface blocked blood permeation both transversely and longitudinally. This autophobic pseudo-dewetting behavior enabled in situ blood concentration and exerted a local dehydration effect, thereby significantly accelerating hemostasis and reducing blood loss [165].

#### 5.1.2. Acceleration and Amplification of the Coagulation Process

Once the coagulation cascade has been successfully initiated, further accelerating key reaction steps—particularly thrombin generation and fibrin formation—is essential for shortening hemostasis time. By directly delivering bioactive macromolecules or constructing platelet-mimetic systems, upstream coagulation processes can be partially bypassed, thereby enabling rapid hemostasis. As the rate-limiting enzyme in the coagulation process, efficient delivery and stable immobilization of thrombin remain major challenges for practical applications. Hou et al. incorporated thrombin into a bacterial cellulose/poly(vinyl alcohol) (BC/PVA) composite sponge through a combination of physical adsorption and covalent immobilization, thereby achieving sustained release while preserving enzymatic activity and ultimately enhancing overall coagulation efficiency [27]. Ma et al. utilized the synergistic interaction between cationic chitosan and thrombin to simultaneously achieve rapid hemostasis and antibacterial activity [166]. Furthermore, Ibne Mahbub et al. developed a thrombin-loaded decellularized liver extracellular matrix (L-ECM)/oxidized cellulose nanofiber (TOCN)/CS nanocomposite system. In this study, insoluble L-ECM was combined with thrombin to construct a bioactive hemostatic matrix, achieving hemostasis times of approximately 71 s in a rat tail-amputation model and approximately 41 s in a liver laceration model, while also demonstrating superior liver tissue repair capability compared with the commercially available Surgicel^®^ product [167]. In addition to direct supplementation with coagulation factors, mimicking platelet function has also emerged as an important research direction. Shuai et al. developed an injectable fibrinogen microsphere system that achieved specific binding to fibrin and targeted aggregation at bleeding sites through conjugation with thrombus-targeting peptides, thereby mimicking the physiological function of natural platelets. In vivo experiments demonstrated that this system formed stable thrombi within approximately 2 min and reduced blood loss by 74% compared with unmodified fibrin microspheres [168]. Moreover, incorporation of platelet-rich plasma (PRP)-derived exosomes not only provides multiple coagulation-related factors but also delivers pro-angiogenic signaling molecules, thereby supporting subsequent tissue repair and regeneration processes [169].

#### 5.1.3. Hemostatic Stabilization and Prevention of Re-Bleeding

Following the achievement of initial hemostasis, the stability of the blood clot directly influences the risk of rebleeding, particularly under conditions associated with hyperfibrinolysis or complex wound microenvironments. Therefore, inhibiting fibrin degradation and maintaining clot structural integrity represent critical subsequent stages of hemostatic therapy. Tranexamic acid (TXA), a classical antifibrinolytic agent, effectively suppresses fibrin degradation by blocking the binding of plasmin to fibrin [170,171]. Tang et al. developed a TXA-loaded catechol-modified hyaluronic acid/carboxymethyl chitosan bicrosslinked hydrogel system capable of achieving sustained local drug release, thereby prolonging hemostatic duration [172]. For deep wounds or cavity hemorrhages, balancing volumetric adaptability with controlled drug-release behavior is particularly important. Wang et al. developed TXA-modified super-expandable microsponges, in which TXA-modified gelatin was immobilized onto highly compressed functionalized chitosan sponges through strong hydrogen-bond interactions. This strategy helped maintain the compressed state of the material while enabling rapid release of stored expansion potential following thermally responsive dissolution. The resulting system achieved more than 11-fold volume expansion within 5 s, continuously released antifibrinolytic agents, and simultaneously provided physical filling of the wound cavity. In both disseminated intravascular coagulation (DIC) and coagulopathy models, this material demonstrated superior hemostatic efficacy compared with bulk sponges and commercially available collagen sponges [173]. Building upon these approaches, multifunctional composite systems have further integrated antifibrinolytic agents with anti-inflammatory regulation or antibacterial activity, thereby simultaneously maintaining clot structural stability and modulating the local wound microenvironment during the late-stage hemostatic phase, ultimately reducing the risk of rebleeding [174,175]. Overall, these strategies enable targeted intervention at different stages of the coagulation cascade and demonstrate considerable potential for applications involving coagulopathy, uncontrolled hemorrhage, and complex traumatic injuries.

### 5.2. Antimicrobial Therapy

For gunshot wounds occurring in harsh environments such as battlefields, infection has become the second leading cause of mortality after hemorrhagic shock. A strong synergistic relationship exists between infection and bleeding: blood and exudate retained within the wound after hemostasis provide favorable conditions for bacterial colonization. Simultaneously, once bacteria form biofilms on the wound surface, acidic metabolites and secreted proteases abnormally activate matrix metalloproteinases (MMPs) and the fibrinolytic system, thereby accelerating fibrin degradation, directly disrupting the initial coagulation network, and ultimately leading to secondary massive hemorrhage [176,177]. Therefore, the development of hemostatic materials with integrated antibacterial activity has become an urgent clinical requirement.

#### 5.2.1. Antimicrobial Agent Loading

During the early stage of infection, localized delivery of antimicrobial agents through hydrogel networks represents a direct and effective strategy for suppressing bacterial proliferation. Although conventional antibiotics such as ciprofloxacin and tobramycin can effectively eliminate pathogens, their relatively narrow antibacterial spectrum makes them prone to inducing bacterial resistance during prolonged use, thereby limiting their clinical applicability [178]. In contrast, spatiotemporally controlled release of metal nanoparticles, metal ions, and naturally derived plant extracts exhibits superior multi-target antibacterial efficacy and improved biosafety. Silver nanoparticles (AgNPs) release Ag^+^ ions that readily penetrate the bacterial peptidoglycan cell wall and form strong coordination interactions with thiol groups (–SH) in bacterial respiratory chain enzymes, leading to irreversible enzyme inactivation. Simultaneously, Ag^+^ interferes with cysteine-containing bacterial proteins, thereby disrupting bacterial DNA replication and transcription processes. In addition, intracellular accumulation of metal ions induces severe oxidative stress, triggering excessive ROS generation and causing sustained damage to pathogenic microorganisms [177,179]. Based on this mechanism, Li et al. employed Artemisia annua extract to green-synthesize AgNPs and incorporated them into hemostatic materials, thereby achieving synergistic enhancement of antibacterial activity and tissue repair [20]. Beyond Ag^+^, other metal ions such as Zn^2+^ and Cu^2+^ also exhibit antibacterial activity and can participate in coagulation regulation by promoting platelet adhesion and accelerating the coagulation process [180,181]. Considering the potential toxicity associated with metallic components, naturally derived bioactive molecules have gradually emerged as important alternative antibacterial agents. Plant essential oils are enriched in highly lipophilic terpenoids and phenolic bioactive compounds capable of inserting into bacterial phospholipid bilayers, thereby disrupting membrane integrity and inducing leakage of intracellular contents [182,183]. Polyphenolic compounds such as TA and epigallocatechin gallate (EGCG) can form multiple hydrogen bonds with bacterial cell wall proteins, thereby interfering with bacterial metabolic activity [184]. Based on these mechanisms, Xin et al. combined natural polysaccharides with drug-loaded microspheres to achieve synergistic antibacterial activity and hemostatic regulation in diabetic wound models [185]. Ding et al. further demonstrated that incorporation of EGCG into hydrogel systems simultaneously enhanced infection control and tissue regeneration [186]. Compared with single-mechanism antibacterial strategies, such multi-pathway chemical interventions exhibit greater stability and adaptability in complex wound microenvironments.

#### 5.2.2. Intrinsic Antimicrobial Activity

Endowing materials with intrinsic antibacterial activity has become a key strategy for achieving long-term anti-infective protection. Natural polymers containing abundant cationic groups, such as chitosan, QCS derivatives, and ε-polylysine (ε-PLL), can strongly interact with negatively charged bacterial cell membranes through electrostatic interactions, thereby inducing membrane depolarization and disrupting membrane integrity to achieve contact-dependent bactericidal activity [178]. Chen et al. developed a triple-network hydrogel based on CMCS and oxidized dextran (ODex), which exhibited broad-spectrum antibacterial activity both in vitro and in vivo while significantly promoting the healing of infected wounds [187]. Similarly, positively charged network systems constructed using ε-polylysine (PLL) have also demonstrated rapid surface sterilization capability [188]. In addition, incorporation of antimicrobial peptides (AMPs) into hydrogel systems provides another effective strategy for sustained antibacterial activity. AMPs possess amphiphilic structures and can directly disrupt bacterial membranes through the formation of transmembrane pores. Because these molecules primarily target membrane structures, they maintain potent bactericidal efficacy while exhibiting favorable biocompatibility [189]. Wang et al. screened and identified the AMP LI5 and further incorporated it into hydrogel systems for hemostatic applications [190]. Overall, contact-dependent antibacterial mechanisms provide hydrogel-based anti-infective systems with a sustained antimicrobial foundation that does not rely solely on controlled release behavior.

#### 5.2.3. Photothermal/Photodynamic Therapy

**Figure 8:**
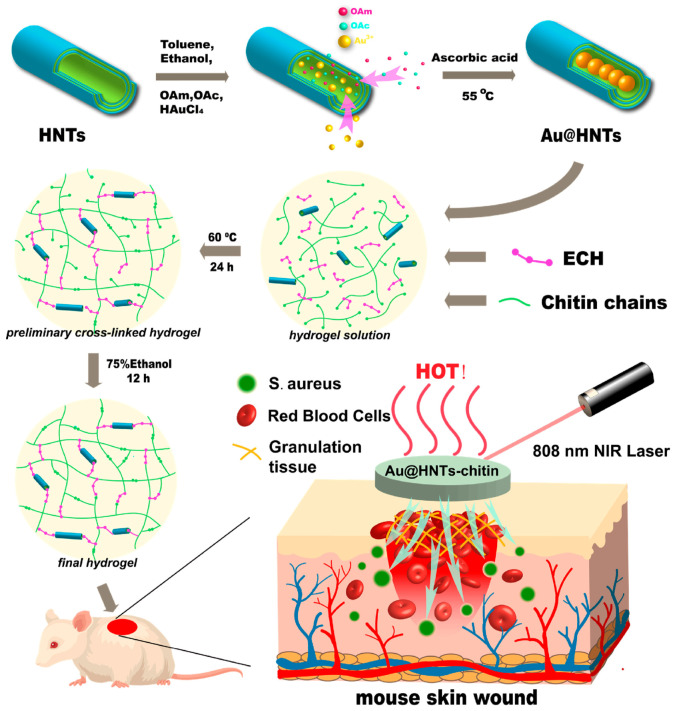
Schematic showing the preparation process and application of Au@HNTs (halloysite clay nanotubes) -chitin hydrogel. Reprinted with permission from ref. [192]. Copyright 2022 Zhao et al. Publishing services by Elsevier B. V. on behalf of KeAi Communications Co. Ltd.

Biofilms are composed of extracellular polysaccharides, proteins, and extracellular DNA (eDNA), which collectively form a dense extracellular polymeric substance (EPS) matrix. This structure not only restricts the penetration of antimicrobial agents but also weakens immune-mediated bacterial clearance. Although the aforementioned antibacterial strategies exhibit good efficacy against planktonic bacteria, they still face substantial limitations in the presence of mature biofilms. Therefore, incorporation of external-field-responsive physical intervention strategies is of considerable significance. Photothermal therapy (PTT) generates localized hyperthermia under near-infrared (NIR) irradiation through the incorporation of photothermal agents such as PDA, gold nanoparticles, or carbon-based nanomaterials into hydrogel systems. This localized thermal effect can directly destroy bacterial structures, induce protein denaturation, and simultaneously accelerate the coagulation process. In the chitosan/polydopamine composite hydrogel developed by Chang et al., PDA served as the NIR-absorbing component and rapidly generated heat under light irradiation, thereby achieving complete eradication of pathogenic bacteria and promoting tissue repair in burn and infected wound models [42]. Similarly, the thermosensitive injectable chitosan-based hydrogel reported by Hou et al. also integrated photothermal-responsive functionality [191]. A composite hydrogel dressing based on halloysite nanotubes (HNTs) and chitin was developed (Figure 8), integrating the advantages of biomacromolecules and clay. HNTs feature a hollow tubular structure with charged SiOx and AlOx surfaces. Au nanoparticles (5–10 nm) were loaded into the HNTs lumen, with oleic acid (OAc) and oleylamine (OAm) serving as stabilizing agents. HAuCl_4_ and ascorbic acid were used to generate Au NPs within the tubes. The Au@HNTs were then mixed with chitin and crosslinked with epichlorohydrin (ECH), forming a flexible hydrogel. ECH forms covalent ether bridges between chitin chains and HNTs, enhancing structural stability and mechanical properties. Ethanol immersion was used to remove residual reagents and may further stabilize the hydrogel network. The Au@HNTs-chitin hydrogel exhibits antibacterial, hemostatic, and wound-healing activity with low cytotoxicity. In vivo studies demonstrated effective inhibition of *Staphylococcus aureus* infection and accelerated wound closure. This design provides a promising strategy for developing high-performance wound dressings from commonly used biocompatible materials [192]. Wu et al. further demonstrated excellent programmable photothermal antibacterial activity using carbon nanotube-based composite sponges [193]. Photodynamic therapy (PDT), in contrast, generates ROS through photoexcitation of photosensitizers, thereby effectively degrading the EPS matrix of bacterial biofilms and inducing oxidative destruction of bacteria. To achieve precise photodynamic targeting of infected sites, He et al. recently developed a protoporphyrin IX (PpIX)-modified chitosan/sodium alginate photodynamic cryogel. In this system, phenylboronic acid (PBA) groups introduced onto the sodium alginate backbone formed dynamic boronate ester bonds with diol-containing structures on bacterial cell walls, thereby enabling specific bacterial targeting and adhesion. Following bacterial immobilization within the gel network, the PpIX photosensitizer generated high local concentrations of ROS upon light irradiation, enabling efficient elimination of planktonic bacteria while simultaneously promoting degradation of mature biofilm structures. Moreover, the macroporous cryogel architecture facilitated rapid blood absorption and concentration, thereby exhibiting highly synergistic effects between photodynamic antibacterial activity and rapid physical hemostasis in infected wound models in vivo [194].

Overall, the anti-infection design strategies of hemostatic hydrogels have evolved from single-agent antimicrobial delivery systems toward multilevel regulatory platforms integrating chemical intervention, contact-dependent antibacterial activity, and external-field responsiveness. Such systems can target both planktonic bacteria and mature biofilms across different temporal stages, while simultaneously improving the infected wound microenvironment to maintain clot structural stability, reduce the risk of rebleeding, and provide favorable conditions for subsequent tissue repair.

### 5.3. Tissue Regeneration

In prehospital emergency care and clinical surgical procedures, acute massive hemorrhage often causes severe local tissue ischemia and hypoxia. Such injuries are highly susceptible to progression toward persistent oxidative stress and excessive inflammatory responses, thereby impairing wound healing [195]. Therefore, the design of hemostatic materials should not only address bleeding control but also eliminate the biochemical damage remaining after hemostasis and provide a favorable microenvironment for tissue regeneration. By integrating functional modules with temporal regulation capabilities into the post-hemostasis phase, these materials can sequentially achieve antioxidant regulation, immune microenvironment remodeling, angiogenesis, and ECM reconstruction, thereby extending their function from simple hemostasis to tissue repair.

#### 5.3.1. Antioxidant Regulation and Inflammation Alleviation

During the early stages of tissue injury and in chronic wounds, ischemia–reperfusion processes can induce a respiratory burst in neutrophils, leading to the excessive generation of ROS, including hydroxyl radicals (·OH), superoxide anions (O_2_·^−^), and hydrogen peroxide (H_2_O_2_). Although low levels of ROS participate in cellular signaling regulation, their sustained accumulation can trigger lipid peroxidation, DNA fragmentation, and protein inactivation, ultimately inducing the apoptosis of fibroblasts and endothelial cells and maintaining the wound in a chronic inflammatory state [196]. Therefore, restoring redox homeostasis represents a prerequisite for effective wound healing. From the perspective of material design, functionalizing the hydrogel network through the incorporation of natural bioactive molecules rich in phenolic hydroxyl groups represents one of the most direct antioxidant strategies. These polyphenolic molecules can react with free radicals through hydrogen atom transfer or single-electron transfer mechanisms to generate stable intermediates, thereby terminating lipid peroxidation chain reactions. In the oxidized chondroitin sulfate/carboxymethyl chitosan hydrogel developed by Zhao et al., coordination nanoparticles (ICPs) composed of proanthocyanidins, berberine, and Fe(III) were incorporated into the hydrogel matrix. Owing to the electron-donating capability of their polyphenolic structures, these nanoparticles effectively alleviated oxidative damage in diabetic wounds [160]. Similarly, Zhou et al. fabricated a multifunctional drug-loading hydrogel (HA@TA-Okra) from hyaluronic acid methacrylate integrated with tannic acid and okra extract, which achieved synergistic antioxidant and bactericidal effects via the two bioactive components. Benefiting from tannic acid-mediated crosslinking, the hydrogel acquired robust mechanical stability, rapid gelation and tunable swelling capacity, thereby realizing localized on-demand drug release, rapid hemostasis, infection suppression, relieved inflammatory response and accelerated full-thickness infected skin regeneration [197]. Compared with antioxidant strategies that rely on molecular consumption, the introduction of nanozymes with enzyme-like catalytic activity provides a sustainable catalytic pathway for ROS scavenging. For example, the Cu/Mn bimetallic nanozyme system developed by Zhang et al. can mimic the cascade catalytic activities of natural superoxide dismutase (SOD) and catalase (CAT), continuously converting ROS into harmless products. This process effectively restores local redox homeostasis and alleviates inflammatory responses through regulation of the NF-κB and JAK–STAT signaling pathways [198]. Furthermore, carbon-based nanomaterials exhibit considerable multifunctionality in antioxidant regulation. Studies have shown that green-synthesized carbon dots (G-CDs) derived from gladiolus extract not only scavenge ROS but also inhibit TLR4/NF-κB-related signaling pathways, thereby reducing the expression of inflammatory factors and accelerating wound healing within a relatively short period [199].

#### 5.3.2. Immune Regulation

Following the alleviation of oxidative stress, the progression of wound healing largely depends on whether the immune system can successfully transition from inflammatory defense to tissue repair. Among these processes, the phenotypic transition of macrophages from the pro-inflammatory M1 phenotype to the pro-repair M2 phenotype is considered a pivotal biological event governing the entry of wounds into the regenerative phase. In chronic wounds, persistent inflammatory stimulation causes macrophages to remain in the M1 phenotype for prolonged periods, leading to the continuous secretion of pro-inflammatory cytokines such as tumor necrosis factor-α (TNF-α) and interleukin-6 (IL-6), which further damage newly formed tissues. Therefore, inducing macrophage polarization toward the M2 phenotype through rational material design has become a core strategy for immune microenvironment remodeling. One important approach involves exploiting specific structural motifs of natural polymers to directly regulate cellular signaling pathways. The tussah silk nanofibrils (ApNFs) developed by Duan et al. contain arginine-glycine-aspartic acid (RGD) sequences. RNA sequencing analysis demonstrated that this structure activated the JAK2–STAT5b and PI3K–Akt signaling pathways while simultaneously suppressing NF-κB-mediated inflammatory responses, thereby promoting macrophage polarization toward the M2 phenotype [200]. This material–cell interaction-based design enables the hydrogel to provide structural support while simultaneously exhibiting immunomodulatory functions. In more complex systems, nanocomposite structures can further achieve synergistic regulation through multiple signaling pathways. For example, core–shell microspheres constructed in one study promoted anti-inflammatory responses and M2 macrophage polarization through several mechanisms, including inhibition of NF-κB/P-JNK-related signaling pathways, reduction of pro-inflammatory factor expression, regulation of the mitochondrial oxidative state, and subsequent activation of the STAT6 signaling pathway [201]. Furthermore, naturally active molecules such as chlorogenic acid can also promote macrophage phenotypic transition and alleviate chronic inflammatory responses through regulation of NF-κB- and JAK–STAT-related signaling pathways [202].

Overall, the essence of this strategy lies in reshaping the immune microenvironment through signaling pathway reprogramming, thereby enabling the wound to transition smoothly from the inflammatory phase to the tissue repair phase and providing a favorable cellular foundation for subsequent tissue regeneration.

#### 5.3.3. Vascular Regeneration and Matrix Reconstruction

Following immune microenvironment remodeling, the final bottleneck in the tissue repair process shifts toward the reconstruction of the vascular network and the ECM. Insufficient blood supply directly impairs cell proliferation and collagen deposition; therefore, angiogenesis plays a decisive role in tissue regeneration.

Owing to their structural similarities to the ECM, hydrogels serve as ideal platforms for the delivery of pro-angiogenic factors. Among these, exosomes, as critical mediators of intercellular communication, can activate endothelial cell functions through paracrine signaling mechanisms. Exosomes derived from mesenchymal stem cells, progenitor cells, blood products, immune cells, and natural sources consistently improved wound healing by enhancing angiogenesis, re-epithelialization, fibroblast proliferation, and extracellular matrix remodeling, while decreasing oxidative stress and chronic inflammation. Mechanistically, these effects were regulated via the stimulation of the PI3K/AKT, ERK/MAPK, STAT3, HIF-1α/VEGF, and Nrf2 signaling, together with the suppression of the AGE/RAGE-regulated ferroptosis and apoptosis [203]. In addition to exosomes, the controlled delivery of growth factors is also essential for vascular regeneration and tissue repair. For example, a basic fibroblast growth factor (bFGF) sustained-release system based on cyclodextrin-mediated host–guest interactions was shown to promote ECM remodeling and angiogenesis through activation of the CXCR4 pathway, significantly improving the healing quality of complex wounds [204]. Compared with biomacromolecular therapeutics, metal ions provide a more stable source of pro-angiogenic signals. For instance, Mg^2+^ and Cu^2+^ can promote cell migration and angiogenesis, whereas Zn^2+^ accelerates microvascular network reconstruction by upregulating vascular endothelial growth factor (VEGF) expression [205,206]. The sustained release of these inorganic signaling factors endows hydrogels with long-term tissue regeneration regulatory capabilities. Overall, the realization of immune regulation and tissue regeneration functions follows a clear stepwise progression. First, microenvironmental homeostasis is restored through antioxidant regulation; subsequently, chronic inflammatory responses are terminated through immune microenvironment remodeling; finally, tissue regeneration is achieved through angiogenesis and ECM reconstruction.

### 5.4. Dynamic Monitoring and Stimuli-Responsive Capabilities

The aforementioned functions, including enhanced hemostasis, antibacterial activity, and tissue regeneration, primarily address how wounds can be treated. However, in practical clinical care, wounds covered by dressings are often difficult to observe directly. Traditional hydrogel systems, which mainly rely on passive release mechanisms, lack the ability to provide real-time feedback regarding internal bleeding, infection, and inflammatory progression. Consequently, clinical decision-making frequently depends on empirical dressing replacement and intermittent assessments. Such delayed information acquisition can readily lead to peak–trough effects during drug delivery and may further cause secondary damage to newly formed tissues and increased risks of infection accumulation. Accordingly, integrating pathological signal monitoring and on-demand response capabilities into natural polymer-based hydrogels has gradually emerged as a key strategy for improving therapeutic outcomes. These designs exploit the sensitivity of natural polymer networks to microenvironmental changes, including variations in pH, ROS, and specific enzyme levels, thereby enabling real-time pathological signal detection and feedback-regulated intervention [207]. It should be emphasized that these functional modules are primarily intended for postoperative monitoring and chronic wound management rather than prehospital emergency care. Their primary objective is to reduce the risks of rebleeding and infection progression while simultaneously promoting tissue regeneration through continuous data acquisition and precise therapeutic intervention.

#### 5.4.1. Visualization and Dynamic Monitoring of Pathological Signals

**Figure 9:**
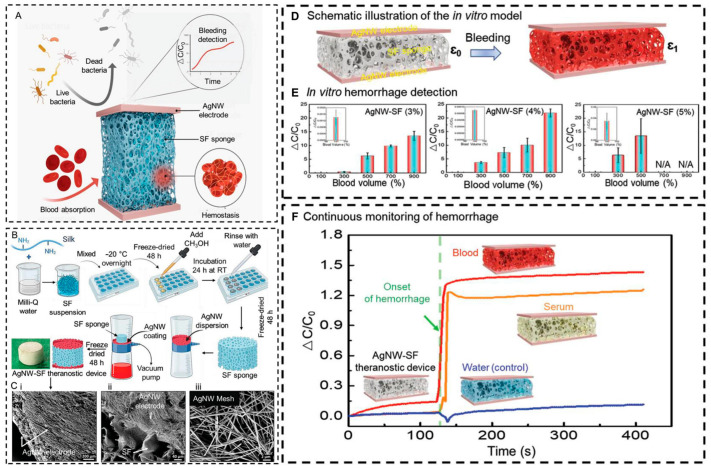
(**A**) Conceptual illustration of the silver nanowire-silk fibroin (AgNW-SF) theranostic device for intelligent hemorrhage management. (**B**) The fabrication process of the AgNW-SF theranostic device. (**C**) Composition characterizations of AgNW-SF theranostic device. (**i**) Top-view SEM image of the AgNW-SF theranostic device. (**ii**) The magnified cross-sectional SEM image reveals a thin AgNW electrode on the surface of the porous SF sponge, as indicated with arrows. (**iii**) Magnified SEM image of AgNWs to confirm their uniformity in diameter and length. (**D**) In vitro monitoring of the hemorrhage with AgNW-SF theranostic device. A capacitance sensor is created by mounting two AgNW electrodes on the top and bottom surfaces of the SF sponge. The device detects bleeding when blood penetrates the SF sponge and modifies the dielectric constant from ε_0_ to the higher value of ε_1_. (**E**) Bleeding detection potential of different concentrations of AgNW-SF devices. (**F**) Continuous monitoring of hemorrhage by distinct variation in capacitance after absorption of blood versus serum and water. Reprinted with permission from ref. [208]. Copyright 2023 Haghniaz et al. Advanced Science is published by Wiley-VCH GmbH.

A wound is essentially a highly dynamic biochemical reaction system, and its physiological state and clinical risks exhibit pronounced temporal variations. Therefore, real-time monitoring of key pathological signals and their conversion into interpretable information represent prerequisites for establishing precision intervention systems. For hemostatic materials, maintaining the stability of the coagulation barrier is of primary importance, making the identification of occult rebleeding particularly critical. To address this challenge, researchers have developed sensing systems based on the unique physicochemical properties of blood. For example, Haghniaz et al. developed an all-in-one AgNW-SF theranostic platform, in which SF is sandwiched between two silver nanowire (AgNW) electrodes to non-enzymatically detect local bleeding and promote wound hemostasis (Figure 9A). The shape-memory SF sponge accelerates clotting, reducing in vitro hemostatic time by ~82%. The device comprises top and bottom AgNW electrodes for conductivity and antibacterial function, and a central SF sponge for hemostasis and capacitive sensing (Figure 9B). AgNW films form a 10–15 µm conductive layer (Figure 9(Ci,ii)), with individual nanowires ~113 nm in diameter and ~24 µm long (Figure 9(Ciii)). As a capacitive sensor, the device enables real-time hemorrhage monitoring (Figure 9D). Blood infiltration increases the dielectric constant of the SF sponge, causing measurable capacitance changes. A 4% SF sponge provides optimal sensitivity and range (Figure 9E). The device distinguishes blood from other fluids, such as serum and water, ensuring accurate detection (Figure 9F). It is antibacterial, biodegradable, and shows no significant inflammatory response, with hemostatic efficacy comparable to commercial hemostats in rat liver models [208]. To further address the risk of postoperative rebleeding after cerebral hemorrhage, Yu et al. developed a hemoglobin (Hb)-responsive implantable DNA hydrogel. Upon rebleeding, Hb molecules in blood are specifically recognized and bound by aptamers within the hydrogel network, triggering hydrogel dissociation. Simultaneously, characteristic optical signals are generated through fluorescence resonance energy transfer (FRET), thereby enabling the detection of deep-tissue rebleeding [209].

After the early risk phase has passed, the focus of wound management gradually shifts toward infection and inflammatory regulation. In infected or chronic wounds, bacterial metabolism frequently reduces the local pH to approximately 5.0–6.5, whereas inflammatory responses are typically accompanied by elevated ROS levels. Based on these pathological changes, embedding pH/ROS-responsive probes into natural polymer networks enables in situ monitoring of microenvironmental variations, which can be intuitively visualized through colorimetric or fluorescence signal changes. Building upon this concept, several studies have further introduced data-driven analytical strategies to reduce human judgment bias during signal interpretation. Jiang et al. developed a conductive hydrogel based on QCS. By training a ResNet34 convolutional neural network using a large dataset of hydrogel colorimetric images, this smart hydrogel system can decode subtle wound pH fluctuations in real time via a smartphone, achieving a classification accuracy of up to 93.25% and enabling highly sensitive early warning of deep infection [210]. Furthermore, the development of conductive hydrogels has endowed wound dressings with electronic-skin-like functionalities. Conductive networks constructed from natural polysaccharides can continuously collect signals associated with mechanical deformation and metabolic activities, thereby enabling real-time monitoring of wound tension variations and local physiological conditions [211]. Overall, these strategies facilitate the conversion of otherwise unobservable pathological processes into interpretable information, thereby providing a rational basis for subsequent on-demand therapeutic intervention.

#### 5.4.2. Pathology-Triggered On-Demand Therapeutic Release

Once pathological signals have been detected, the key challenge in designing such functional systems lies in implementing on-demand therapeutic interventions in response to microenvironmental changes. Compared with traditional sustained-release systems, stimulus-responsive networks can convert pathological signals into triggers for drug release, thereby enabling dynamic matching between therapeutic intervention and disease progression. Taking postoperative rebleeding following cerebral hemorrhage as an example, relying solely on signal warning systems is insufficient for timely intervention. Ideally, functional materials should simultaneously recognize rebleeding-associated signals and initiate the release of procoagulant agents, thereby shortening the therapeutic response time. Recent studies have shown that biochemical molecules enriched in fresh blood, such as hemoglobin (Hb) and thrombin, as well as local shear-force alterations induced by blood flow, can serve as endogenous triggering signals for responsive hemostatic systems. Yu et al. developed an Hb-responsive in situ implantable DNA hydrogel composed of Hb aptamers cross-linked with complementary DNA strands and loaded with deferoxamine mesylate (DFO) for the treatment of postoperative intracerebral rehemorrhage. Upon rebleeding, rapid Hb enrichment triggered detectable optical signals through Hb capture, endowing the hydrogel with a “self-diagnosis” capability. Simultaneously, continuous Hb binding induced gradual disintegration of the hydrogel network, enabling on-demand DFO release without disrupting physiological iron-dependent functions. The released DFO inhibited neuronal ferroptosis and significantly reduced hematoma volume in a mimic postoperative rehemorrhage model. Nevertheless, because DFO mainly served as a model therapeutic agent in this study, the broader applicability of Hb-responsive DNA hydrogels as delivery platforms for other therapeutics still requires further investigation [209]. In infected wounds, acidic microenvironments and elevated ROS levels represent typical pathological triggering signals. Networks constructed based on dynamic covalent bonds can remain stable under physiological conditions while undergoing selective cleavage in pathological microenvironments, thereby triggering controlled drug release. Chen et al. achieved hierarchical release of epigallocatechin gallate (EGCG) by introducing boronic acid groups into chitosan and constructing a dual-responsive system with oxidized polysaccharides. Under acidic conditions, the network initially dissociated, whereas elevated ROS levels further accelerated the release process [212]. Similarly, Sun et al. exploited the acid responsiveness of Schiff base bonds to achieve precise controlled release of hydrogen sulfide (H_2_S)-donor nanoparticles, effectively avoiding tissue toxicity associated with burst gas release [213]. In addition to chemical stimuli, temperature also serves as an important physical trigger. Natural polymers exhibiting a lower critical solution temperature (LCST) remain in a solution state at room temperature but rapidly undergo phase transition and form hydrogels at physiological temperature, while simultaneously enabling drug release. The thermoresponsive chitosan derivatives developed by Cai et al. achieved rapid phase transition without requiring additional crosslinking agents, thereby combining excellent hemostatic performance with stimulus-responsive properties [214].

To further improve response selectivity, enzyme-specific recognition mechanisms can also be incorporated into hydrogel systems. In chronic wounds, the expression levels of matrix metalloproteinases (MMPs), particularly MMP-9, are significantly upregulated. By integrating peptide crosslinkers or enzyme-responsive substrates with specific recognition sequences into natural polysaccharide scaffolds, selective responsiveness toward target enzymes can be achieved. The MMP-9-responsive hydrogel developed by Meng et al. underwent degradation and released exosomes only under inflammatory conditions, thereby enabling precise pathology-triggered drug delivery [215]. Overall, these strategies transform pathological alterations into material response signals to achieve condition-dependent drug release, thereby endowing the therapeutic process with adaptive characteristics and significantly enhancing the timeliness and specificity of therapeutic intervention.

#### 5.4.3. External-Field Regulation and Interdisciplinary Extensions

Although response systems based on endogenous microenvironmental cues possess a certain degree of adaptability, their triggering processes are still constrained by the heterogeneous spatiotemporal distribution of pathological signals. To further improve regulatory precision, recent studies have increasingly incorporated external-field regulation and multimodal control strategies into hydrogel systems. Among various external-field regulation approaches, ultrasound has attracted considerable attention owing to its excellent tissue penetration capability and non-invasive characteristics. By incorporating piezoelectric nanomaterials such as barium titanate, acoustic energy can be converted into electrical signals, thereby regulating cellular behaviors and inflammatory responses. Studies have demonstrated that microcurrents generated under ultrasonic stimulation can modulate PI3K/Akt-related signaling pathways and promote macrophage polarization [216]. Simultaneously, under ultrasonic irradiation, polyoxometalate-based systems can catalyze the conversion of ROS, thereby enabling dynamic regulation of the oxidative state [217]. Magnetically responsive systems provide an alternative strategy for remote regulation in deep tissues. Magnetoelectric coupling materials can convert external magnetic signals into localized electrical stimulation, thereby enabling programmed regulation during different stages of wound healing [218]. In addition, magnetically guided microneedle systems can achieve precise positioning and targeted hemostatic intervention within complex wound cavities [219]. The introduction of self-powered systems has further expanded the application potential of these functional modules. Hydrogels integrated with triboelectric nanogenerators (TENGs) can convert human motion into electrical signals, thereby enabling continuous electrical stimulation without an external power supply while simultaneously regulating the drug release process [220,221].

In recent years, with the rapid development of interdisciplinary research, hydrogel systems have gradually been integrated with microfluidic technologies and flexible electronic components to construct more sophisticated monitoring and regulation platforms. For example, a smart bandage system integrated with a microcontroller employs biopolymer hydrogels as skin-interfacing electrodes to achieve continuous multiplexed monitoring of skin impedance and temperature. Through autonomous decision-making by the microcontroller, the system can precisely apply electrical stimulation to the wound and achieve on-demand detachment after treatment completion [222]. Another class of microfluidic flexible systems, termed iCares, utilizes superhydrophobic/superhydrophilic Janus membranes to enable spontaneous unidirectional transport of wound exudate without external driving forces. Combined with electrochemical sensor arrays, these systems enable continuous quantitative monitoring of indicators such as ROS, pH, and temperature, thereby significantly improving the stability and reliability of long-term wound monitoring [223]. Overall, through the incorporation of external physical fields and multimodal integration strategies, hydrogel systems have evolved from passive biomaterials into tunable integrated platforms, providing new technical pathways for precise intervention in complex wound environments.

## 6. Conclusions and Outlook

Although natural polymer-based hydrogels have achieved substantial experimental progress in hemostasis and wound repair, their translation from laboratory research to clinical application remains constrained by multiple challenges, including raw material sourcing, toxicological safety, engineering scale-up, and regulatory approval pathways. As the interdisciplinary integration of materials science, bioelectronics, and artificial intelligence continues to advance, future research should focus on overcoming these critical bottlenecks to promote the development of hydrogel systems toward higher precision and improved clinical applicability.

### 6.1. Overcoming Raw Material Barriers

Although conventional natural polysaccharides and animal-derived proteins, such as collagen, exhibit excellent biocompatibility, their molecular weight distribution and reactive crosslinking sites are highly susceptible to variations arising from extraction batches. Moreover, these materials often present issues associated with endotoxin residues, potential immunogenicity, and pathogen transmission risks. In recent years, recombinant human proteins and synthetic biology strategies have gradually emerged as promising alternative approaches. For example, recombinant human type III collagen (rhCol III) and functionalized synthetic peptides can substantially reduce immunological risks while enabling precise amino acid sequence engineering, including the introduction of matrix metalloproteinase (MMP)-responsive sites and the programmed arrangement of cell adhesion motifs. Consequently, material fabrication strategies are shifting from conventional extraction and post-modification processes toward programmable manufacturing approaches based on expression regulation. Nevertheless, such systems still face practical challenges related to large-scale production efficiency and cost control.

### 6.2. Improving Full Life-Cycle Toxicological Evaluation

Current studies primarily focus on improving hemostatic performance by enhancing local procoagulant activity, such as through the introduction of high-density positive charges, sustained calcium ion release, or the incorporation of exogenous thrombin. However, under conditions involving deep hemorrhage or minimally invasive procedures, excessive procoagulant activity may introduce systemic risks. Once active degradation fragments or charged particles enter the bloodstream, they may disrupt physiological coagulation homeostasis, thereby inducing distal thrombosis, which in severe cases may further progress to disseminated intravascular coagulation (DIC). Simultaneously, degradation products generated from complex chemical modifications, including cationic oligomers or fragments released during dynamic bond cleavage, may also trigger inflammatory responses or cytotoxic effects. Therefore, future evaluation systems should extend beyond the assessment of hemostatic efficacy alone and establish a comprehensive analytical framework encompassing degradation behavior, immune responses, hemocompatibility, and systemic effects on the coagulation process.

### 6.3. Advancing Intelligent and Digital Closed-Loop Systems

Wound management is gradually evolving from passive wound coverage toward dynamic monitoring and active regulation. Future hemostatic hydrogels are expected to integrate with bioelectronic devices to construct multifunctional platforms combining microfluidic regulation and multi-parameter sensing capabilities. Through continuous monitoring of key indicators, including pH, temperature, metabolites, and inflammatory factors, and by integrating artificial intelligence (AI) and machine learning algorithms to dynamically evaluate wound status and predict healing trends, therapeutic interventions—such as triggered drug release or external physical stimulation—could be implemented before the onset of infection or rebleeding. Simultaneously, such systems may further integrate with telemedicine platforms, thereby improving the continuity and precision of wound management. However, their practical application is still constrained by challenges related to sensor stability, power supply strategies, and overall system complexity.

### 6.4. Balancing Functional Complexity and Clinical Feasibility

During clinical translation, regulatory requirements exert a direct influence on material design strategies. Although the integration of multiple functional modules, including antibacterial activity, immunomodulation, and smart sensing, can enhance therapeutic performance, their practical necessity should be carefully evaluated according to specific clinical needs. In future hydrogel development, priority should be given to a limited set of essential properties tailored to particular indications. For acute prehospital trauma, rapid hemostasis and robust wet adhesion are paramount, whereas complex intelligent modules may provide limited additional benefit. In contrast, sustained anti-infective and tissue-regenerative functions remain highly justifiable for chronic wounds and infected wound environments. To realize these advanced functionalities, hydrogel systems often become increasingly complex. While the incorporation of diverse nanocomponents or sophisticated dynamic networks may achieve optimal laboratory performance, it can severely impede engineering scalability and make it difficult to maintain batch-to-batch reproducibility during large-scale manufacturing. Furthermore, increased material and system complexity substantially raises the challenges associated with regulatory approval. Once a material system incorporates drugs or bioactive components, it generally requires more stringent regulatory pathways, including Premarket Approval (PMA) or De Novo classification, thereby prolonging development timelines and increasing overall costs. Therefore, future material design should strive to achieve a rational balance between functional integration and system complexity. Minimizing component diversity and simplifying fabrication procedures may improve manufacturability, regulatory feasibility, and eventual clinical adoption. Meanwhile, cost-effectiveness and strong environmental adaptability are particularly important considerations for emergency applications in resource-limited settings.

### 6.5. Comparative Translational Potential of Hemostatic Platforms

When identifying the most clinically promising hydrogel platforms, translational potential should be evaluated holistically through the synergistic interplay of material source, structural design, and functional complexity. As summarized in Table 4, among the various systems, particle-based hydrogels, particularly self-gelling powders, and porous hydrogels currently exhibit the highest translational readiness. Their clinical promise is fundamentally supported by effective integration across all three dimensions. These systems primarily utilize well-characterized natural polymers with minimal chemical modification at the material level, rely on mature and scalable manufacturing techniques such as spray-drying or lyophilization at the structural level, and focus on essential physical occlusion mechanisms rather than complex pharmacological interventions at the functional level. This combination contributes to excellent dry-state shelf stability, straightforward terminal sterilization, and relatively streamlined regulatory approval pathways, including FDA 510(k) clearance. Conversely, platforms with a high degree of sophistication often encounter substantial translational bottlenecks. Fibrous hydrogels, despite their excellent biomimetic functions, remain challenged by the scalable manufacturing of nanofibrous architectures and the batch-to-batch variability associated with natural protein precursors. Furthermore, multicrosslinked or multinetwork hydrogels and nanocomposite hydrogels, while offering exceptional mechanical resilience and intelligent stimulus-responsive functionalities, currently demonstrate lower immediate clinical feasibility. Their extensive material hybridization complicates Good Manufacturing Practice (GMP) scale-up, while the incorporation of active nanocomponents or dynamic bonds can restrict available sterilization strategies. More importantly, the integration of multiple advanced functions may transform these systems into high-risk combination products, necessitating extensive toxicological validation and more stringent PMA processes. Therefore, emphasizing the most translatable platforms highlights a key principle for future development: maximizing clinical efficacy while minimizing the collective complexity associated with material composition, structural design, and functional integration.

### 6.6. Establishing a Standardized Evaluation System

At present, the evaluation of hemostatic materials still relies predominantly on standardized small-animal models, the outcomes of which often fail to accurately reflect complex clinical scenarios. Future studies should incorporate large-animal models, such as pigs and sheep, while simultaneously simulating clinically relevant conditions, including coagulopathy, anticoagulant therapy, high-shear blood flow environments, and infection, in order to improve the translational relevance and generalizability of experimental findings. Furthermore, direct comparative studies between newly developed materials and currently approved clinical products, such as HemCon^®^, Celox^®^, and Fibrin glue, should be systematically conducted to provide more convincing evidence regarding their therapeutic advantages. Only when these materials demonstrate clear superiority under complex pathological conditions can they establish a robust foundation for subsequent clinical translation and practical application.

## Figures and Tables

**Table 1:** Material design strategies for natural polymer-based hemostatic hydrogels.

Material	Hemostatic Mechanism	Advantages	Limitations	Modification Strategies	References
Chitosan	Protonated amino groups enrich erythrocytes and platelets via electrostatic interactions and promote the assembly and activation of coagulation factors and plasma proteins on fiber surfaces	The only naturally cationic polysaccharide, possessing broad-spectrum antibacterial activity and procoagulant function, biodegradable in vivo	Poor solubility at physiological pH, pronounced mechanical brittleness, excessive gelation upon high water absorption	Carboxymethylation, quaternization, hydrophobic alkylation grafting, tannic acid complexation, zwitterionic charge balancing	[25,37,38,39,40,41,42,43,44,45,46,47,48,49,50,51,52,53,54,55,56]
Cellulose	Rapidly absorbs blood fluid to concentrate coagulation components; surface negative charges trigger contact activation of intrinsic coagulation factor XII	The most abundant natural polymer, high crystallinity, excellent chemical and mechanical stability, low cost	Lack of endogenous cellulase may provoke foreign body granuloma; conventional oxidized products exhibit strong acidity that damages tissue and inhibits thrombin activity, delaying healing	TEMPO-mediated selective oxidation, carboxymethylation, calcium ion exchange, in situ dopamine polymerization, ionic or polyphenolic composite modification	[57,58,59,60,61,62,63,64,65]
Alginate	Absorbs fluid physically and releases calcium ions which serve as coagulation factor IV to accelerate the coagulation cascade	Rich in guluronic acid blocks enabling rapid in situ gelation with multivalent cations, low toxicity and negligible immunogenicity	Pure calcium-crosslinked networks readily disintegrate via ion exchange in physiological fluids, exhibiting poor mechanical stability and resistance to blood flow erosion	Schiff base dynamic covalent crosslinking, reinforcement with ultralong hydroxyapatite nanowires, construction of zinc-mediated multi-coordination networks	[66,67,68,69,70]
Hyaluronic acid	Physically fills and seals wounds; specifically binds CD44 receptors on cell surfaces to facilitate migration and tissue repair	Outstanding cytocompatibility, carries biorecognition motifs such as CD44, serving as an ideal platform bridging hemostasis and subsequent tissue regeneration	Excessive hydrophilicity causes interfacial lubrication and slippage under blood flow; the molecular backbone lacks active moieties to initiate the coagulation cascade	Grafting of phenylboronic acid to form dynamic boronate ester bonds for wet adhesion; conjugation of adenosine diphosphate to actively activate platelets	[71,72,73]
Starch	Achieves mechanical tamponade and blood concentration through extreme fluid absorption and swelling	Abundant hydrophilic hydroxyl groups confer high swelling capacity, excellent biosafety, and extremely low material cost	Biologically inert with no inherent charge or cell recognition sites; rapid degradation by endogenous amylases limits long-term barrier function	Quaternization to introduce positive charge, serotonin grafting to activate platelets, aldehyde or catechol grafting to enhance interfacial adhesion, construction of reversible boronate ester or acylhydrazone dynamic networks	[74,75,76,77,78,79,80,81]
Collagen and gelatin	The Arg-Gly-Asp sequences and GFOGER motifs specifically mediate platelet adhesion, activation, and aggregation	Naturally contains cell-adhesive recognition motifs and matrix metalloproteinase cleavage sites, enabling complete bioabsorption	Physical gels disintegrate at body temperature; uncontrolled degradation in protease-rich wounds; animal-derived sources carry risks of immunogenicity and pathogen transmission	Methacrylation for photocrosslinking, grafting of acetylcysteine or catechol groups, specific peptide modification, polysaccharide-mediated mild crosslinking	[82,83,84,85,86,87,88,89,90]
Silk fibroin	Forms a β-sheet structure to seal wounds and promotes platelet pseudopodia extension and activation	Exceptional mechanical strength and shape recovery, tunable degradation rate, significantly lower immunogenicity than mammalian collagen	Spontaneous gelation relying on natural conformational transition is extremely slow; lacks the capacity for covalent bonding on wet interface to withstand arterial pressure	Dual side-chain modification with methacrylate and dimethylamino groups for rapid photocrosslinking and charge-mediated synergistic effects complexation with tannic acid to enhance wet adhesion	[91,92,93,94,95,96,97,98]
Fibrin	Mimics the terminal stage of physiological coagulation, forming a fibrin network clot catalyzed by thrombin	Perfectly recapitulates the architecture of native blood clots, high specific surface area facilitates cell infiltration and angiogenesis	Pure fibrin networks have low mechanical strength and are rapidly degraded by plasmin; clinical use depends on exogenous thrombin with high cost and risk of distal thrombosis	Sequential crosslinking to embed gelatin methacryloyl photosensitive networks for mechanical reinforcement; calcium ion tuning to induce supramolecular self-assembly into pseudo-fibrin structures	[99,100,101,102,103,104,105]

**Table 2:** Structural design of hemostatic hydrogels based on natural polymers.

Hydrogel Structure Type	Formation Mechanism and Synthesis	Key Hemostatic Features	Limitations	References
Porous hydrogels	Freeze-drying, phase separation, gas foaming, or ice-templating creates 3D interconnected pores; directional freeze-casting yields oriented microchannels.	High porosity/surface area enables rapid fluid absorption and blood concentration; swelling occludes irregular wound cavities; aligned channels lower flow resistance and concentrate coagulation factors.	Trade-off between absorption rate and mechanical stability; lack of quantitative structure–function design rules.	[106,107,108,109,110,111,112,113,114,115,116]
Particle-based hydrogels	Emulsion polymerization, microfluidics, spray drying, or inverse emulsion crosslinking produces microspheres/powders; self-gelling powders crosslink and aggregate upon contact with blood.	Discrete particles allow injectable, conformal filling; swelling-induced jamming provides physical compression; self-gelling powders form cohesive adhesive networks; porous microspheres internally concentrate coagulation factors.	Prone to blood flow erosion and migration; reassembled networks lack bulk mechanical continuity; in vivo clearance fate remains unclear.	[117,118,119,120,121,122,123,124,125,126,127,128,129]
Fibrous hydrogels	In situ supramolecular self-assembly (e.g., peptide hydrogen bonding, π–π stacking) triggered by body fluids; ex vivo performed via electrospinning, polyelectrolyte complexation, or β-sheet induction.	Nanofibrous ECM-mimetic topography promotes platelet adhesion/activation; in situ assembly rapidly forms a physical barrier; preformed Janus structures enable directional exudate drainage.	Assembly kinetics slower than blood flow may fail to provide mechanical sealing; limited volumetric expansion restricts deep-cavity filling.	[130,131,132,133,134,135,136,137,138,139,140,141,142,143]
Multicrosslinked/Multinetwork hydrogels	Dual physical–chemical crosslinking or interpenetrating polymer networks; dynamic reversible bonds (Schiff base bonds, boronate ester bonds, ionic coordination) dissipate energy and enable self-healing.	Physical bonds dissipate energy, chemical bonds maintain integrity → high strength/toughness; dynamic networks tolerate cyclic deformation (e.g., heart, major arteries); simultaneously improves wet adhesion and burst pressure.	Complex degradation matching among components; high crosslinking density may mask bioactive sites.	[144,145,146,147,148,149,150,151,152]
Nanocomposite hydrogels	Blending, in situ growth, or electrostatic assembly of inorganic nanofillers (layered silicates, hydroxyapatite, carbon nanomaterials, metal-coordinated NPs) into polymer networks.	Nanofillers reinforce modulus and fatigue resistance; silicates activate intrinsic coagulation via charged surfaces; carbon-based fillers add photothermal/electroconductivity; hydroxyapatite promotes osteogenesis.	Risk of nanoparticle release and distal embolization; aggregation/deactivation in the hemorrhagic microenvironment; incomplete toxicological and metabolic data.	[153,154,155,156,157,158,159,160]

**Table 3:** Advanced functionalities of natural polymer-based hemostatic hydrogels.

Functional Category	Specific Strategy	Core Mechanism and Effect	References
Coagulation modulation and hemostatic enhancement	Coagulation cascade initiation enhancement: loading inorganic minerals such as kaolin, zeolite, and mesoporous bioactive glass	Surface negative charges contact activation of coagulation factor XII; porous structures enrich factors X/V and accelerate thrombin burst formation, shortening initiation time	[161,162,163,164,165]
	Coagulation cascade amplification: delivering thrombin, cationized chitosan, or constructing platelet-mimetic microparticles	Direct supplementation of rate-limiting enzymes or mimicking platelet membrane function bypasses upstream cascades to rapidly catalyze fibrin formation, suitable for thrombocytopenia	[166,167,168,169]
	Hemostatic stabilization and prevention of rebleeding: loading antifibrinolytics such as tranexamic acid	Inhibits plasminogen binding to fibrin, blocks fibrin degradation, maintains clot mechanical stability, and reduces rebleeding rate	[170,171,172,173,174,175]
Antimicrobial Therapy	Antibacterial agent loading: Ag, Zn, Cu nanoparticles/ions, plant essential oils, and polyphenolic natural molecules	Metal ions disrupt respiratory chain enzymes and DNA; essential oils/polyphenols compromise membrane integrity. Multi-target bactericidal action with natural molecules exhibiting concurrent antioxidant activity	[176,177,178,179,180,181,182,183,184,185,186]
	Inherent antibacterial activity: cationic polymers such as quaternized chitosan (QCS), ε-polylysine, and antimicrobial peptides	Electrostatic adsorption disrupts bacterial cell membranes, induces depolarization and intracellular leakage, providing a sustained contact-killing barrier	[187,188,189,190]
	Photothermal therapy (PTT) and photodynamic therapy (PDT): incorporating polydopamine, gold nanoparticles, carbon materials, or protoporphyrin IX photosensitizers	Photothermal effects generate localized hyperthermia to destroy bacteria; photodynamic action produces reactive oxygen species to degrade biofilm matrix. Spatiotemporally controllable and effective against mature biofilms	[191,192,193,194]
Immunomodulation and tissue regeneration	Antioxidant and anti-inflammatory: polyphenol networks, nanozymes, carbon dots, and other ROS-scavenging systems	Hydrogen atom or single electron transfer terminates radical chain reactions; biomimetic SOD/CAT cascade catalysis eliminates ROS, restoring redox homeostasis	[160,195,196,197,198,199]
	Immune reprogramming: RGD motif modification, core–shell microspheres, chlorogenic acid, and other active molecules	Activates JAK2-STAT5b/PI3K-Akt pathways, inhibits NF-κB, and induces macrophage polarization from M1 pro-inflammatory to M2 pro-regenerative phenotype, terminating chronic inflammation	[200,201,202]
	Angiogenesis and matrix remodeling: exosomes, controlled-release growth factors, delivery of Mg/Zn/Cu ions	Regulates PI3K/AKT, ERK/MAPK, STAT3, HIF-1α/VEGF, Nrf2 pathways and inhibits AGE/RAGE-mediated ferroptosis and apoptosis; metal ions upregulate VEGF expression, promoting microvascular network reconstruction and collagen deposition	[203,204,205,206]
Dynamic Monitoring and stimuli-responsive capabilities	Pathological signal visualization: hemoglobin-responsive DNA hydrogels, pH/ROS probes, electronic skin systems	Hemoglobin triggers aptamer conformational change generating optical signals; pH/ROS induce colorimetric changes; conductive networks acquire mechanical deformation and metabolic electrical signals	[207,208,209,210,211]
	Stimuli-responsive controlled release: pH/ROS/enzyme-sensitive dynamic bond networks, thermoresponsive phase transition systems	Pathological microenvironments trigger cleavage of Schiff base, boronate ester, and other dynamic bonds or induce LCST-type polymer phase transition, matching drug release dynamics with disease progression	[209,212,213,214,215]
	Externally controlled actuation and theranostic integration: ultrasound-driven piezoelectric, magnetic navigation, triboelectric nanogenerators, microfluidic flexible systems	Ultrasound/magnetic fields remotely activate piezoelectric/magnetoelectric materials to generate electrical stimulation modulating cellular behavior; self-powered systems enable autonomous operation; microfluidics achieve exudate management and multi-indicator electrochemical sensing	[216,217,218,219,220,221,222,223]

**Table 4:** Comparative assessment of the translational potential of natural polymer-based hemostatic hydrogel platforms.

Platform	Raw Material Readiness	Manufacturing Maturity	Sterilization Compatibility	Regulatory Complexity	Commercialization Potential
Particle-based hydrogels	High	High	High	Low	High
Porous hydrogels	High	High	High	Low	High
Fibrous hydrogels	Moderate	Moderate	Moderate	Moderate	Moderate
Multicrosslinked/Multinetwork hydrogels	Moderate	Low	Low	High	Moderate-Low
Nanocomposite hydrogels	Moderate	Low	Low	High	Low

Note: Translational readiness was qualitatively assessed according to raw material accessibility, manufacturing maturity, sterilization compatibility, regulatory complexity, and overall commercialization potential.

## Data Availability

No new data were created.
